# Efficient Production and Isolation of 3‐Acetamido‐5‐Acetylfuran from N‐Acetyl‐D‐Glucosamine within Protic Ionic Liquids

**DOI:** 10.1002/open.202500094

**Published:** 2025-02-25

**Authors:** Emma L. Matthewman, Jonathan Sperry, Cameron C. Weber

**Affiliations:** ^1^ School of Chemical Sciences University of Auckland 23 Symonds Street Auckland 1010 New Zealand; ^2^ MacDiarmid Institute for Advanced Materials and Nanotechnology Wellington New Zealand

**Keywords:** Amines, Biomass valorization, Carbohydrates, Ionic liquids, Chitin

## Abstract

The transformation of chitin and its monomer N‐acetylglucosamine (NAG) to high‐value organonitrogen chemicals has attracted increasing interest, with 3‐acetamido‐5‐acetylfuran (3A5AF) proposed as a versatile platform chemical. The preparation of 3A5AF from NAG has relied on high boiling organic solvents, ionic liquids (ILs) or deep eutectic solvents (DES). While these methods have met with some success, the isolation of 3A5AF and recycling of the solvent remains problematic for non‐IL methods whereas most IL methods utilize inherently expensive aprotic ILs with substantial environmental footprints. This study details the preparation of 3A5AF in more cost‐effective chloride‐based protic ILs (PILs) with lower synthetic footprints than conventional ILs. Maximum yields of 42.5 %, 51.5 % and 57.0 % of 3A5AF were afforded in 1,8‐diazabicyclo[5.4.0]undec‐7‐ene chloride ([DBU]Cl), tripropylammonium chloride ([TPA]Cl) and tributylammonium chloride ([TBA]Cl) respectively with 2 eq. B(OH)_3_ at 150 °C. The 3A5AF formed was readily isolated by simple solvent extraction, avoiding column chromatography, with selected systems displaying good recyclability and scalability. E‐factor calculations revealed that the PIL methodology produced substantially less waste than approaches for the production of 3A5AF from molecular solvents and DES, highlighting that PILs are suitable solvents for the sustainable production of 3A5AF.

## Graphical abstract

This paper presents a facile method for the conversion of N‐acetylglucosamine (NAG) to 3‐acetamido‐5‐acetylfuran (3A5AF), a potential platform chemical, within protic ionic liquid systems. Reaction optimization, 3A5AF isolation, green metrics and the recyclability and scalability of selected systems are discussed.

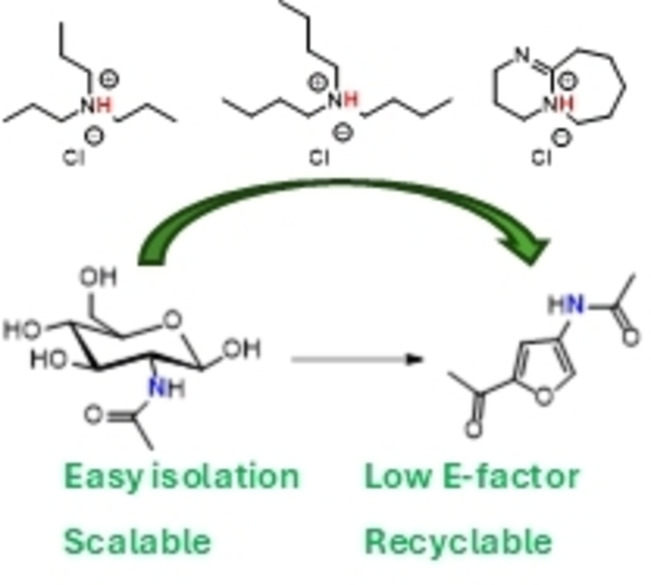

## Introduction

Amid increasing global concerns surrounding the rapid depletion of natural resources and production of greenhouse gases, recent research has shifted its focus towards the utilisation of biogenic renewable feedstocks.^[1]^ Whilst considerable progress has been made in the transformation of lignocellulosic biomass, significantly less research has been conducted on chitin; a structural biopolymer comprising 15–40 % of crustacean exoskeletons.^[2]^ In addition to being the second most plentiful biopolymer, chitin is also the most abundant and accessible source of biologically fixed nitrogen on Earth. Considering nitrogenous compound synthesis currently requires ammonia derived from the carbon‐intensive Haber‐Bosch process, the direct transformation of chitin into useful chemical products and intermediates is highly desirable.^[3]^

**Figure 1:**
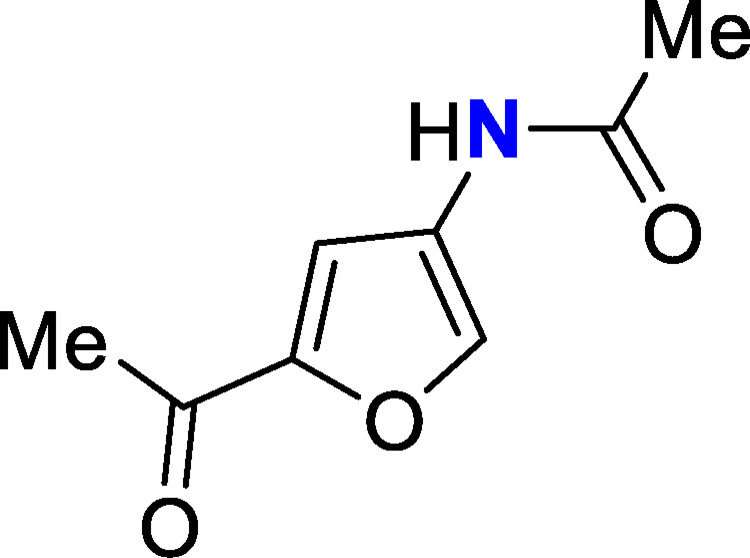
Structure of 3A5AF (biogenic nitrogen in blue).

A diverse range of valuable nitrogenous chemicals can be derived from chitin and its constituent monomer N‐acetyl‐D‐glucosamine (NAG), including amino sugars, chromogens and heterocyclic pyrazines.[^3^, ^4^, ^5^] Of particular significance is 3‐acetamido‐5‐acetylfuran (3A5AF; Figure 1); a potentially important platform chemical which can provide access to pharmaceutical compounds and other structurally complex organonitrogen products.[^6^, ^7^, ^8^, ^9^, ^10^, ^11^] Further to preserving the biologically fixed nitrogen present in chitin, 3A5AF also exhibits a substitution pattern which is challenging to achieve using existing synthetic techniques. The ability to obtain 3A5AF directly from chitin therefore represents a promising step towards more sustainable chemical synthesis.

3A5AF was first prepared by Franich et al. through the pyrolysis of NAG at 400 °C, although only a 2 % yield was obtained.^[12]^ The catalytic dehydration of NAG in dipolar aprotic solvents has proven to be a more effective approach. Omari et al. reported a 58 % yield of 3A5AF in dimethylacetamide (DMA) with B(OH)_3_ and NaCl under microwave irradiation at 220 °C.^[13]^ DMA has also been used alongside other Cl‐containing salts, often in conjunction with B(OH)_3_, to prepare 3A5AF from NAG in yields ranging from 38–63 %.[^14^, ^15^, ^16^, ^17^] Other dipolar aprotic solvent approaches include the use of *N*‐methyl‐2‐pyrrolidone (NMP) and dimethylformamide (DMF), with the reaction promoted again through the use of Cl‐containing salts affording yields in the range 29–68 %, with the highest yielding reaction involving a combination of CaCl_2_, B_2_O_3_ and a pyridinium chloride ionic liquid (IL) as additives.[^18^, ^19^, ^20^, ^21^, ^22^, ^23^] Ji et al. identified γ‐valerolactone (GVL) in conjunction with HCl and NH_4_SCN as a viable reaction system for 3A5AF, with yields of 75.3 % being observed.^[24]^ In this case, the NH_4_SCN served to assist both the solubilisation and conversion of NAG. The preparation of 3A5AF from chitin in a one‐step procedure has also been achieved by Chen et al., affording 7.5 % yield using B(OH)_3_ and LiCl as additives in NMP.^[25]^ Despite these methods generating 3A5AF in reasonable quantities; environmental concerns regarding the utilisation of toxic organic solvents, harsh reaction conditions and significant waste generation owing to the poor solubility of NAG in many of these solvents has prompted the exploration of neoteric solvents.

One promising class of alternative solvents are ILs; low melting salts which are recognised as potential alternatives to common molecular solvents.[^26^, ^27^] Generally composed of weakly associated organic cations and inorganic anions, ILs possess several favourable attributes such as negligible vapour pressure and low flammability. These promising characteristics, along with the ability to tune their physiochemical properties through ion selection, have contributed to the extensive investigation of their use in chemical transformations. The direct conversion of chitin to 3A5AF in ILs was first conducted by Chen et. al in 2015.^[28]^ It was found that Cl‐based ILs were required to facilitate 3A5AF formation, with the highest yield of 6.2 % obtained in 1‐butyl‐3‐methylimidazlium chloride ([C_4_C_1_im]Cl) with the addition of B(OH)_3_ and HCl. Improved 3A5AF yields were afforded using chitin pre‐treatment, with ball‐milled chitin affording the highest yield of 20.2 %.^[29]^ ILs have also been used to prepare 3A5AF from NAG, with Drover et al. reporting a 3A5AF yield of 60 % in [C_4_C_1_im]Cl with B(OH)_3_ using microwave heating at 180 °C.^[30]^ Although the IL [C_4_C_1_im]Cl has been found to be an excellent reaction medium for the valorization of NAG and chitin, it requires 22 synthetic steps to prepare from raw materials. This increases its inherent cost of production and leads to significant and unavoidable waste generation from its preparation, limiting its potential as a green solvent.^[31]^ Pereira et al. have very recently used machine learning approaches to enable 3A5AF to be prepared in 70 % yield using a combination of p‐toluenesulfonic acid, NaCl and B(OH)_3_ in tetrapropylammonium chloride.^[32]^ Given the reported melting point of tetrapropylammonium chloride exceeds the reaction temperature, it is likely that the acid and salt form a eutectic to enable the reaction to proceed, although this was not explicitly discussed.

Deep eutectic solvents (DES) have also been utilised for the formation of 3A5AF, albeit at markedly reduced yields. The first report highlighted a 1 : 1:0.5 mole ratio mixture of choline chloride, PEG200 and boric acid which led to a 18.3 % yield of 3A5AF from NAG at 180 °C.^[6]^ Recently, a 1 : 5 : 1 molar ratio of proline: glycerol: lactic acid was used as a solvent to produce 36 % 3A5AF at 120 °C after 2 h without the use of any further additives, although this was formed alongside 20 % chromogen III.^[33]^

Protic ionic liquids (PILs) are a subset of ILs which are formed by proton transfer between a Bronsted acid and Bronsted base.^[34]^ Owing to their simple and inexpensive preparation, there is growing interest in the utilisation of PILs as reaction media. PILs have been successfully employed as solvents and catalysts for several biomass transformations, including the conversion of cellulose to furfural^[35]^ and levulinic acid^[36]^, as well as the synthesis of biodiesel from crude oil.^[37]^ While protic chloride salts have been used as additives in organic solvents for 3A5AF preparation,^[15]^ the use of PILs as reaction solvents has not been previously studied. Since PILs have the potential to overcome the high cost and intrinsic multistep syntheses associated with aprotic ILs, with reduced complexity compared to the mixtures found in DES, they represent viable alternative solvent candidates for 3A5AF formation.

Herein, the conversion of NAG to 3A5AF in a range of PILs containing chloride anions will be discussed. The effects of substrate and additive loadings and reaction temperature on 3A5AF formation were investigated alongside the recyclability and scalability of selected PILs.

## Results and Discussion

### Solvent Screening

**Figure 2:**
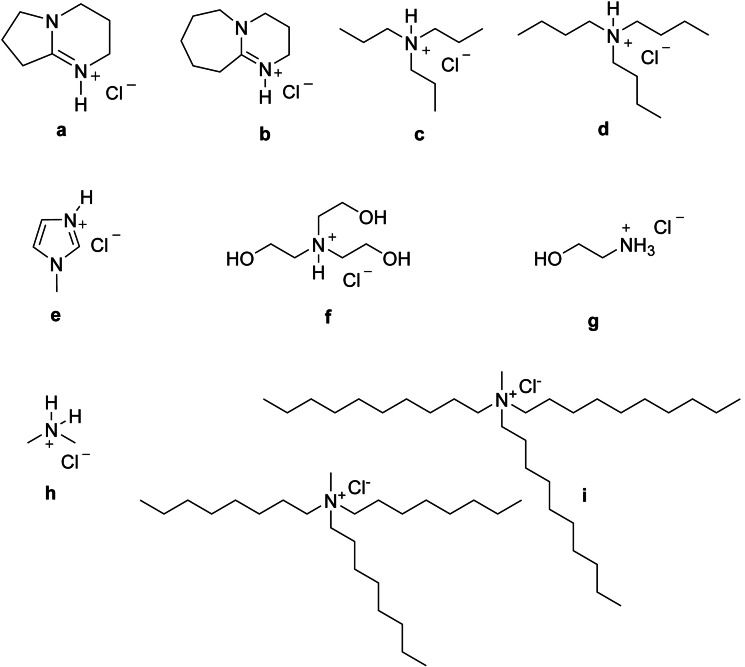
Structures of PILs explored in this study: (a) 1,5‐diazabicyclo[4.3.0]non‐5‐ene chloride ([DBN]Cl), (b) 1,8‐diazabicyclo[5.4.0]undec‐7‐ene chloride ([DBU]Cl), (c) tripropylammonium chloride ([TPA]Cl), (d) tributylammonium chloride ([TBA]Cl), (e) 1‐methylimidazolium chloride ([C_1_im]Cl), (f) triethanolammonium chloride ([TEtA]Cl), (g) ethanolammonium chloride ([EtA]Cl), (h) dimethylammonium chloride ([DMA]Cl, (i) Aliquat 336 (A336).

Prior studies have reported that chloride‐containing ILs and/or additives are usually required for the formation of 3A5AF from both chitin and NAG.[^29^, ^30^] The importance of Cl^−^ has been linked to both its ability to disrupt the hydrogen‐bonding network within chitin, and to promote the dehydration of furan intermediates to afford 3A5AF. Thus, it was of interest to explore the effects of Cl^−^‐based PILs on the conversion of NAG to 3A5AF. While most Cl^−^‐based salts possess melting points above the optimal working temperature for 3A5AF preparation (120–180 °C),^[34]^ some suitable PILs were identified. ILs and the abbreviations used herein are shown in Figure 2. To gain insight into the effect of PILs on this transformation, an aprotic ammonium chloride salt, A336, composed of a mixture of tri‐n‐octylmethylammonium chloride and tri‐n‐decylmethylammonium chloride, was also explored.

To determine the effectiveness of these PILs, the formation of 3A5AF from NAG was first attempted for 2 h at 150 °C with 200 mol % B(OH)_3_, relative to NAG (Table 1). Due to the high melting points of [TEtA]Cl and [DMA]Cl, reactions performed using these PILs were instead conducted at 180 °C for 1 h, with [DBU]Cl used under both conditions as a comparison.

**Table 1:** Yields of 3A5AF obtained in PILs at 150 °C. Reactions were performed with 5 wt % NAG relative to the PIL, and 200 mol % B(OH)_3_ relative to NAG. Reported errors are standard deviations obtained from replicate experiments.

PIL	3A5AF Yield (%)	NAG Conversion (%)
	120 min	
[EtA]Cl	0.15±0.02^[a]^	100^[a]^
[DBN]Cl	3.8±3.2^[a]^	94.9±2.3^[a]^
A336	12.2±1.7^[a]^	43.1±3.0^[a]^
[C_1_im]Cl	18.5±0.08^[a]^	100^[a]^
[TPA]Cl	48.8±4.6^[a]^	88.6±4.3^[a]^
[TBA]Cl	50.5±1.2^[a]^	97.3±2.2^[a]^
[DBU]Cl	42.5±1.4^[a]^	86.6±0.5^[a]^
[DBU]Cl	22.1±0.8^[b]^	93.5±2.0^[b]^
[DMA]Cl	4.4±0.2^[b]^	83.5±1.4^[b]^
[TEtA]Cl	6.0±1.1^[b]^	23.7±4.5^[b]^

[a] Reaction conditions: 0.05 g NAG, 0.028 g B(OH)_3_, 1 g IL, 150 °C, 2 h. [b] Reaction conditions 0.05 g NAG, 0.028 g B(OH)_3_, 1 g IL, 180 °C, 1 h.

From Table 1, it is apparent that the 3A5AF yield increased in the order [EtA]Cl<[DMA]Cl≈[DBN]Cl≈[TEtA]Cl<A336<[C_1_im]Cl<[DBU]Cl<[TPA]Cl<[TBA]Cl. This trend indicates that in general protic aliphatic unfunctionalized ammonium or amidinium PILs perform better than aprotic or functionalized IL systems. In all cases, the major product that could be identified was 3A5AF although some potential byproducts such as glucosamine (Glu) are not readily identifiable by the HPLC method used. While attempts were made to quantify these products, difficulties were encountered with the reliability of these methods (see Supporting Information for further details).

The extremely low yield of 3A5AF observed for the [EtA]Cl system was accompanied by complete NAG conversion, indicating that the reaction was limited by side reactions rather than the inherent reactivity of NAG. The formation of polyborate‐ethanolamine complexes between EtA and B(OH)_3_ has been reported under relatively mild conditions, which may affect the reactivity of B(OH)_3_.^[38]^ However, NMR analysis did not indicate any discernible reaction between [EtA]Cl and B(OH)_3_ (Figure S6). Hence, this suggests the formation of alternative NAG hydrolysis products, although none were readily identifiable based on the HPLC analysis.

While structurally similar to [EtA]Cl, [TEtA]Cl led to a higher, albeit still poor, 3A5AF yield of 6.0 % but with a notably lower NAG conversion of only 23.7 %. This was likely due to solubility constraints, with unreacted, undissolved NAG recovered at the end of the [TEtA]Cl reaction. Both [EtA]Cl and [TEtA]Cl have been previously used with some success as additives for 3A5AF formation, with Zang et al. reporting 3A5AF yields of 20.2 % and 51.2 % following the addition of [EtA]Cl and [TEtA]Cl respectively. However, in these reactions both ILs were used as 2 w/v% additives rather than as solvents and had lower measured pH values than would be expected for the pure ILs (1.04 and 3.97, for [EtA]Cl and [TEtA]Cl respectively), indicating a potential role of residual HCl in the reactivity.^[15]^

[DBN]Cl afforded high NAG conversion but poor and highly variable 3A5AF yields, in contrast with the structurally similar [DBU]Cl that provided higher yields with marginally lower NAG conversion. A potential source of this discrepancy was an unidentified synthesis impurity in the DBN, which was able to be reduced but not completely removed through distillation of DBN following literature purification procedures (Figure S7).^[39]^ It was noted that reactions in [DBN]Cl prepared with purified DBN gave slightly higher, but still poor, yields of 3A5AF suggesting that the impurity affected the reaction outcome.

The aprotic IL A336 afforded a 3A5AF yield of 12.2 % after heating at 150 °C for 2 h, with a relatively low NAG conversion of 43.1 %. NAG solubility was not a factor in this reaction which indicates that the aprotic cation hinders the reactivity of NAG. In comparison, the protic IL [C_1_im]Cl led to complete NAG conversion although 3A5AF yields of only 18.5 %, which suggests the formation of hydrolysis byproducts such as Glu rather than the desired 3A5AF. Similar reactivity is likely to have occurred for [DMA]Cl, although direct comparison is complicated by the higher melting point and hence reaction temperature used.

The highest yields of 3A5AF were observed for the aliphatic PILs [TPA]Cl and [TBA]Cl, affording yields of 48.8 % and 50.5 % respectively, alongside [DBU]Cl which led to a yield of 42.5 % after 2 h at 150 °C. Prior investigations into the preparation of 3A5AF from NAG have focused on ILs with aprotic heterocyclic aromatic cations, particularly imidazolium‐based ILs.^[30]^ The high 3A5AF yields obtained here from [TPA]Cl, [TBA]Cl and [DBU]Cl demonstrate that low‐melting chloride salts containing protic aliphatic ammonium cations or amidinium cations can also facilitate efficient 3A5AF formation, with yields comparable to the aprotic IL systems.

The PILs that possess high melting points required a high reaction temperature (180 °C) to allow for the PILs to remain liquid. However, issues with NAG solubility and reduced 3A5AF formation were observed. While the lower melting PILs facilitated improved 3A5AF formation compared to [DMA]Cl and [TEtA]Cl, it appears that it is not a sufficient condition for effective 3A5AF formation. The minimal 3A5AF formation observed in [EtA]Cl suggests that cation functionalization may introduce cross‐reactivity issues, and the low yields obtained in the [DBN]Cl systems indicates the purity of the PIL plays a crucial role in 3A5AF preparation.

From the results obtained, it is apparent that protic chloride salts allowed for improved 3A5AF formation and NAG conversion compared to the representative aprotic system (A336). This suggests that cations which possess strong hydrogen‐bond donating properties have a favourable impact on the conversion of NAG to 3A5AF. The formation of 3A5AF is proposed to proceed through a series of dehydration steps following the ring closure of NAG.^[30]^ Thus, it is reasonable to suggest that PILs can effectively promote the dehydration of reaction intermediates through hydrogen‐bonding interactions. Similar findings have been obtained in prior studies on the effect of [C_4_C_1_im]^+^ on the conversion of NAG to 3A5AF,^[30]^ and while [C_4_C_1_im]Cl is an aprotic IL it possesses considerable hydrogen‐bond donating ability from the C−H in the 2‐position on the imidazolium ring.^[40]^

### Reaction Optimisation

**Figure 3:**
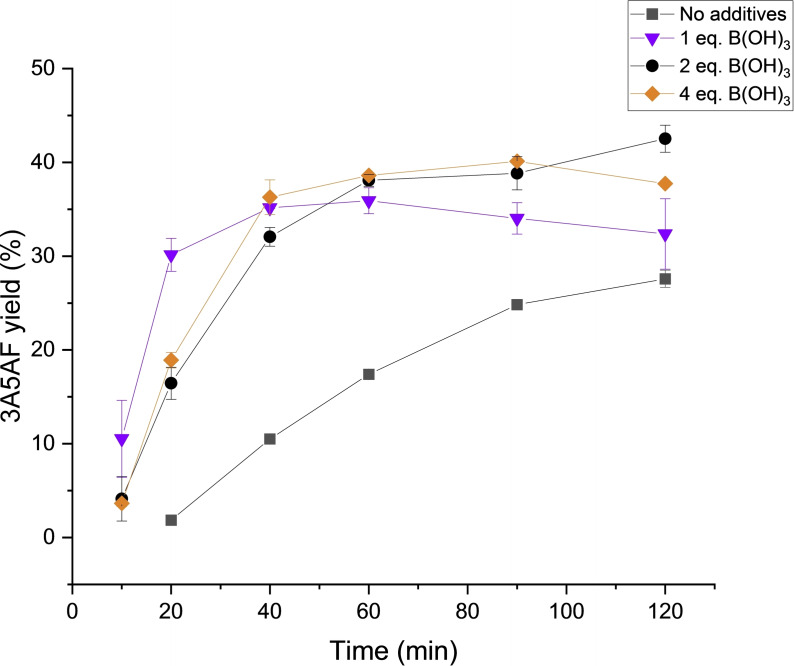
Yields of 3A5AF obtained in [DBU]Cl with different B(OH)_3_ loadings at 150 °C. Reactions were performed with 5 wt % NAG (relative to [DBU]Cl) in 1 g [DBU]Cl. Reported errors are standard deviations obtained from replicate experiments.

**Figure 4:**
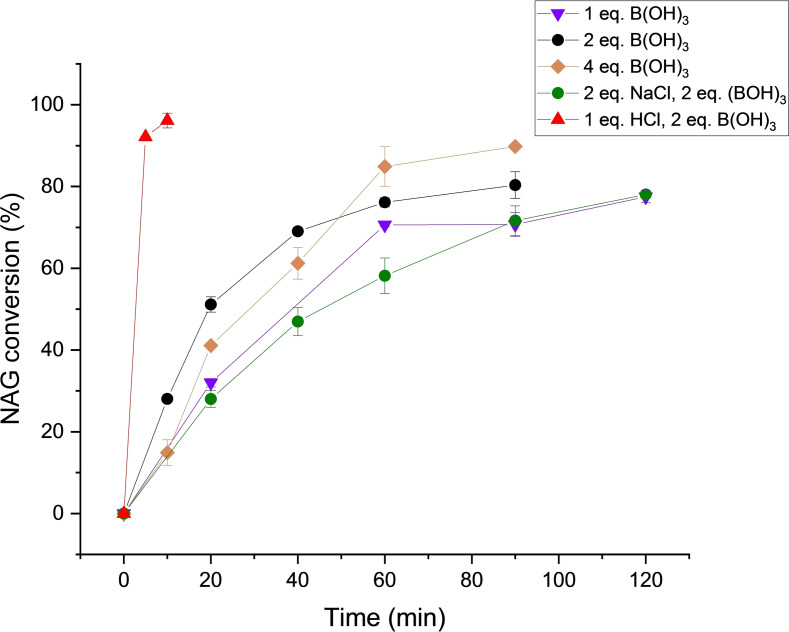
Conversion of NAG in the presence of different additives in [DBU]Cl. Reported errors are standard deviations obained from replicate experiments.

The initial solvent screening investigations revealed the best performing PILs were [DBU]Cl, [TPA]Cl and [TBA]Cl. In efforts to improve 3A5AF yield, the optimisation of reaction conditions was performed in [DBU]Cl. The effect of B(OH)_3_ on reaction outcome was explored at 150 °C (Figure 3). In the absence of added B(OH)_3_ the yield of 3A5AF increased steadily over the course of 2 h, affording a maximum yield of 27.6 %. The yields obtained are comparable to those afforded previously in [C_4_C_1_im]Cl without additives at 180 °C (25.5.%), although the literature yield was obtained after 1 h.^[30]^ 3A5AF formation was then explored with varying B(OH)_3_ loadings (100 mol %, 200 mol % and 400 mol % relative to NAG) at 150 °C. As anticipated, the addition of B(OH)_3_ afforded higher 3A5AF yields, with maximum yields at each B(OH)_3_ concentration of 35.9 %, 42.5 % and 36.8 % with 100 mol %, 200 mol % and 400 mol % B(OH)_3_ respectively. The conversion of NAG in the 100 mol % and 200 mol % B(OH)_3_ systems was approximately 78 %, whilst a conversion above 85 % was observed for the 400 mol % B(OH)_3_ system (Figure 4). The increased NAG conversion afforded with the highest B(OH)_3_ loading suggests excess acid may facilitate formation of other hydrolysis byproducts, as no increase in yield was observed. Given the highest 3A5AF yield was afforded with 200 mol % B(OH)_3_, this system was investigated further.

**Figure 5:**
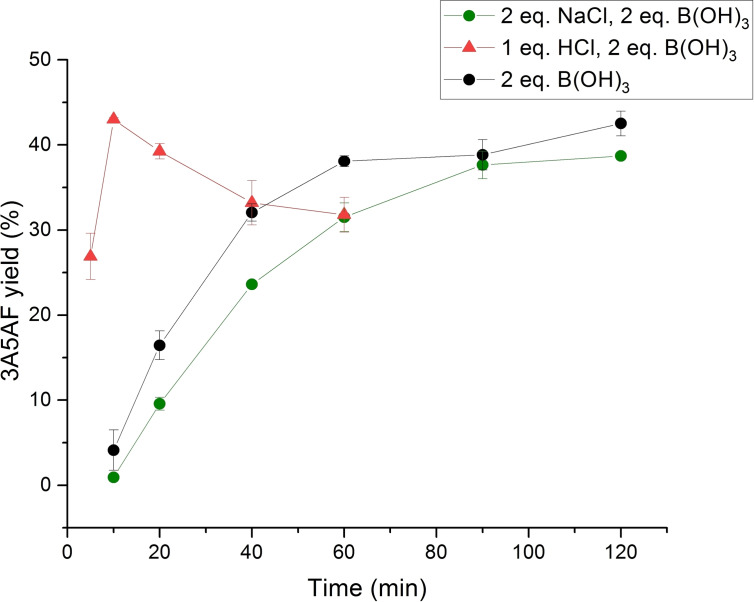
Yields of 3A5AF obtained in the presence of HCl and NaCl at 150 °C. Reactions were performed with 5 wt % NAG (relative to [DBU]Cl), 200 mol % B(OH)_3_ (relative to NAG) and 100 mol % HCl or 200 mol % NaCl (relative to NAG). Reported errors are standard deviations obtained from replicate experiments.

The presence of Cl^−^ is known to enhance the transformation of NAG into 3A5AF in both ILs and organic solvents. A broad range of Cl^−^‐based additives have been explored previously, including acids and metal chlorides. Prior results have revealed that direct chitin conversion benefits from the combination of HCl and B(OH)_3_, whilst group I and II metal chlorides are the most effective additives when NAG is used as the feedstock.[^13^, ^25^, ^28^] To ascertain the effect of using an additional Cl^−^ additive in our [DBU]Cl systems, reactions were performed with HCl and NaCl as representative Cl^−^‐based additives. The yields of 3A5AF obtained with different HCl and NaCl loadings using a fixed mole ratio of 2 : 1 B(OH)_3_/NAG are illustrated in Figure 5.

The incorporation of HCl into the reaction system had a distinct effect on the kinetic profile of the reaction. The yield of 3A5AF reached a maximum of 45.4 % after 10 min, then continually decreased over the remainder of the reaction. The reduction in yield with prolonged reaction time is likely due to product degradation in the presence of excess acid. Conversion of NAG was also almost complete (>95 %) within 10 min (Figure 4). No improvement in yield was observed upon NaCl addition in the [DBU]Cl system, with a maximum 3A5AF yield of 35.9 % and a NAG conversion of 85 % afforded with 200 mol % NaCl, relative to NAG. This suggests that the Cl^−^ within the PIL itself is sufficient to promote 3A5AF formation, with no impact from further Cl^−^ addition.

**Figure 6:**
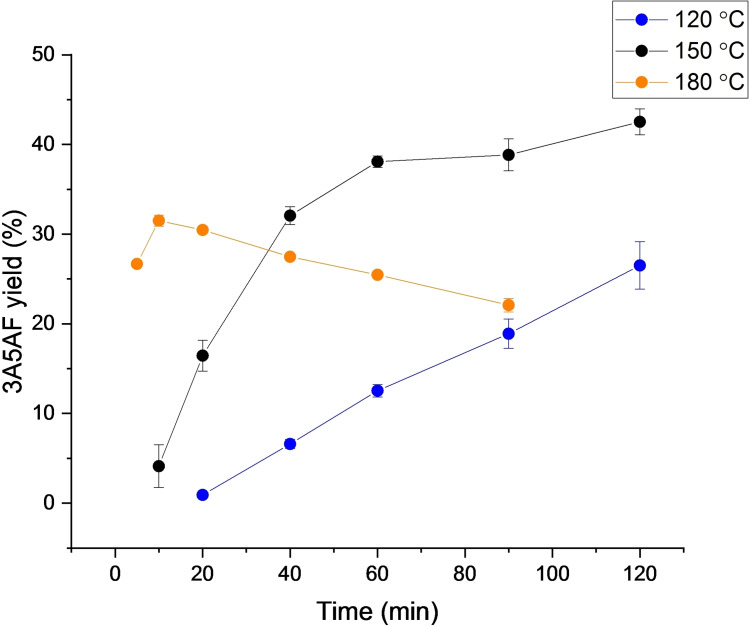
Yields of 3A5AF obtained at different temperatures in [DBU]Cl. Reacions were performed with 5 wt % NAG (relative to [DBU]Cl) and 200 mol % B(OH)_3_ (relative to NAG). Reported errors are standard deviations obtained from replicate experiments.

To gain insight into the effect of reaction temperature, reactions were performed using the optimised B(OH)_3_ loading (200 mol %) at 120 °C and 180 °C. As shown in Figure 6, increasing the reaction temperature resulted in more rapid 3A5AF formation, but also increased product degradation. At 180 °C, a maximum yield of 31.5 % was obtained after 10 min, which decreased at prolonged reaction time. 33.6 % 3A5AF was obtained after 2 h at 120 °C. While it appears that the yield was still increasing after 2 h, the conversion of NAG had reached 78 % and the increased experimental error and reduced rate of change suggests it would be unlikely to reach a higher maximum yield than at 150 °C even if left for a longer time. Based on the maximum yield obtained and product stability under the reaction conditions, 150 °C was deemed to be the optimal reaction temperature.

### Solvent Selection and NAG Loading

**Figure 7:**
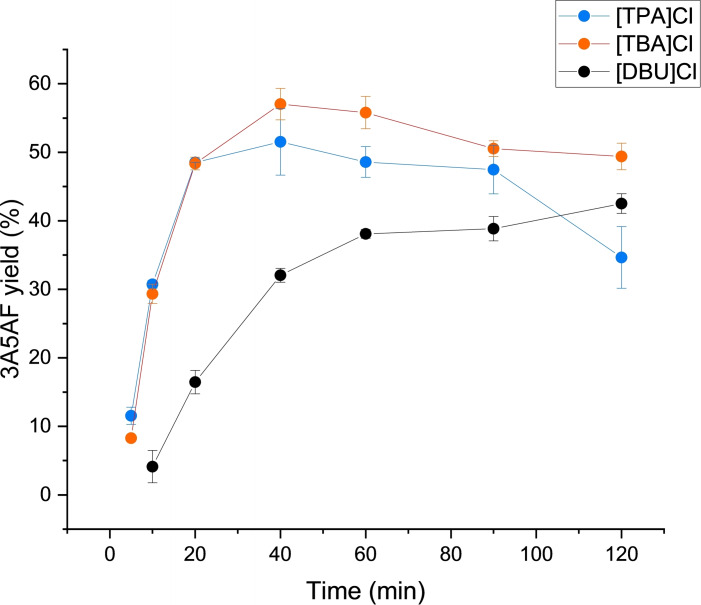
Yields of 3A5AF obtained in [TPA]Cl, [TBA]Cl ad [DBU]Cl at 150 °C. Reactions were performed with 5 wt % NAG (relative to the PIL) and 200 mol % B(OH)_3_ (relative to NAG). Reported errors are standard deviations obtained from replicate experiments.

Following identification of the optimal reaction conditions (150 °C and 2 eq. B(OH)_3_), the effect of time on 3A5AF formation was explored for [DBU]Cl, [TPA]Cl and [TBA]Cl. As shown in Figure 7, [TPA]Cl and [TBA]Cl afforded improved and more rapid 3A5AF formation compared to [DBU]Cl, with maximum 3A5AF yields of 51.5 % and 57.0 %, respectively, after 40 min. The yield of 3A5AF then slowly decreased as the reaction progressed in both PILs, indicative of product degradation. The rate of degradation was slightly faster in [TPA]Cl, which could be due to the slightly higher acidity of this PIL (Table S3).

It was of interest to determine if the NAG loading affected the reaction outcome. Since 3A5AF degradation is observed at prolonged reaction times in both [TPA]Cl and [TBA]Cl, the reactions conducted with 10 wt % NAG were performed for 1 h at 150 °C. The results in Table 2 demonstrate a comparable but slightly decreased yield for [TPA]Cl and [TBA]Cl systems with increased NAG loading, whereas no effect could be observed for [DBU]Cl. Based on the results obtained, a NAG loading of 10 wt % was employed for subsequent investigations as this reduces the volume of solvent required for a given mass of product with minimal impact on the resultant yield.

**Table 2:** Maximum 3A5AF yields obtained using different NAG loadings. Reactions were performed in 1 g IL with 200 mol % B(OH)_3_. Reported errors are standard deviations obtained from replicate experiments.

PIL	NAG Loading (wt %)	Maximum 3A5AF Yield (%)	NAG conversion (%)
[DBU]Cl	5	42.5±1.4	86.6±0.5
[DBU]Cl	10	40.2±1.5	85.5±0.1
[TPA]Cl	5	48.6±2.3	88.6±4.3
[TPA]Cl	10	40.7±2.9	89.7±1.2
[TBA]Cl	5	55.8±2.4	97.3±2.2
[TBA]Cl	10	48.6±0.5	98.9±0.7

The 3A5AF yields observed are similar to those afforded in organic solvents with the addition of chloride‐based salts, including [Gly]Cl,^[14]^ NH_4_Cl,^[23]^ and [TEtA]Cl,^[15]^ although lower than the recent report in GVL with NH_4_SCN and HCl additives.^[24]^ The utilisation of these PILs resulted in enhanced 3A5AF formation compared to literature methods employing ChCl‐based DESs, both as additives and solvents[^6^, ^16^] and are slightly lower but comparable to those afforded in DMA (62 %) and [C_4_C_1_im]Cl (60 %) systems using microwave heating at 220 °C and 180 °C respectively.[^13^, ^30^] In many of these cases, the product isolation was not attempted or required the use of column chromatography or similar techniques that generate substantial solvent waste, hence the isolation of 3A5AF from these PIL systems was explored.

### 3A5AF Isolation

To develop a green approach, a simple ethyl acetate (EtOAc) solvent extraction procedure was explored and optimised. Following the reaction, 3A5AF isolation was achieved by dissolving the reaction mixture in water, then extracting 3A5AF into EtOAc. For the [DBU]Cl system, the EtOAc layer was washed with 0.1 M NaOH, then water and the EtOAc evaporated to afford 3A5AF in a yield of 36.7 %. The high purity of the isolated 3A5AF was confirmed by ^1^H NMR (Figure S8).

The isolation of 3A5AF from [TPA]Cl and [TBA]Cl was first attempted using this EtOAc solvent extraction method, without the use of 0.1 M NaOH. Whilst 3A5AF was partially isolated, a significant amount of each PIL partitioned into the EtOAc layer. For the [TPA]Cl system, the EtOAc layer was washed with 0.1 M HCl to remove residual amine, affording an isolated yield of 32.8 % with no residual solvent. Residual [TBA]Cl was unable to be removed with the HCl washing step, thus other organic solvents were explored. The utilisation of diethyl ether (Et_2_O) reduced the partitioning of [TBA]Cl in the organic layer, allowing 3A5AF to be extracted with an isolated yield of 34.8 % (Table 3). In efforts to establish a greener method, 2‐methyltetrahydrofuran (2‐MeTHF), methyl isobutyl ketone (MIBK) and cyclopentyl methyl ether (CPME) were explored; however, the PIL solution partitioned into 2‐MeTHF and MIBK, and 3A5AF was found to be sparingly soluble in CPME. Due to the challenges associated with isolating 3A5AF from [TBA]Cl without the use of column chromatography, this PIL system was not explored further.

**Table 3:** HPLC and isolated 3A5AF yields obtained from [DBU]Cl, [TPA]Cl and [TBA]Cl using liquid‐liquid extraction.

PIL	Extraction Solvent	HPLC 3A5AF Yield (%)	Isolated 3A5AF Yield (%)
[DBU]Cl	EtOAc	44.8	36.7
[TPA]Cl	EtOAc	43.8	32.6
[TBA]Cl	Et_2_O	48.9	34.8

### Recyclability and Scalability

The recyclability of the [DBU]Cl and [TPA]Cl systems was explored. Following the extraction of 3A5AF using the previous procedure, [DBU]Cl was regenerated by evaporating the water under reduced pressure and reused by either: (1) adding another equivalent of NAG, or (2) adding NAG and an additional 200 mol % B(OH)_3_ relative to the added NAG. 3A5AF yields remained relatively consistent after successive cycles without the addition of B(OH)_3_ (Table 4), while the incorporation of both NAG and B(OH)_3_ led to more significant reductions in yield (Table S4). This is likely due to the degradation of 3A5AF induced by excess B(OH)_3_, as demonstrated in Figure 3. These results also indicate that the B(OH)_3_ recycled from the original reaction mixture remined active for the transformation after the extraction process. Given the results obtained with [DBU]Cl, the recyclability of [TPA]Cl was assessed without additional B(OH)_3_, and similar results were obtained (Table 5). For [TPA]Cl an initial increase and subsequent decrease in HPLC and isolated yields were observed which may be due to the incomplete isolation of 3A5AF from the reaction mixture using the extraction procedure, with slightly reduced reaction efficiency after the second recycle.

**Table 4:** HPLC and isolated yields of 3A5AF obtained in successive cycles in [DBU]Cl. Reaction conditions: 0.10 g NAG, 0.056 g B(OH)_3_, 1 g [DBU]Cl, 150 °C, 1 h.

Reaction cycle	HPLC 3A5AF yield (%)	Isolated yield (%)
1	41.3	36.6
2	41.6	36.7
3	34.3	29.8

**Table 5:** HPLC and isolated yields of 3A5AF obtained in successive cycles in [TPA]Cl. Reaction conditions: 0.10 g NAG, 0.056 g B(OH)_3_, 1 g [TPA]]Cl, 150 °C, 1 h.

Reaction cycle	HPLC 3A5AF yield (%)	Isolated yield (%)
1	43.8	32.6
2	48.8	37.7
3	39.1	28.8

It is worth noting the total weight of IL decreased slightly after 3 reaction cycles in each PIL. This was due to slight solubility of [DBU]Cl and [TPA]Cl in EtOAc, meaning a portion of PIL was extracted into EtOAc in each 3A5AF extraction procedure. This small loss in PIL may account for the moderate reduction in 3A5AF yields observed after 3 cycles. Further optimisation of the extraction procedure to limit loss of [DBU]Cl and [TPA]Cl could improve the reaction outcome for both systems.

Preliminary studies towards scaling up a PIL reaction system were performed in [DBU]Cl. The reactant and solvent amounts were increased 50‐fold, and optimised reaction conditions used. As illustrated in Table 6, similar 3A5AF yields were obtained compared to the 1 g reactions, which indicates this [DBU]Cl system possesses good scalability. Some reduction in isolated yield was observed, which may be due to the proportional solvent volumes for the extraction step being reduced due to both the aim of minimising solvent use and practical constraints. Following product extraction, the recyclability of the scaled‐up reaction system was assessed. Table 6 shows similar 3A5AF yields were afforded upon reusing the [DBU]Cl for successive cycles without the use of additional B(OH)_3_. The slight increase in yield observed in the second cycle is likely due to residual product remaining in the IL from the preceding cycle.

**Table 6:** HPLC and isolated yields of 3A5AF obtained after 3 successive cycles for the scaled‐up reaction. Reaction conditions: 5.0 g NAG, 2.8 g B(OH)_3_, 50 g [DBU]]Cl, 150 °C, 1 h.

Reaction cycle	HPLC 3A5AF yield (%)	Isolated yield (%)
1	36.9	26.6
2	39.3	27.7
3	31.0	25.2

The direct transformation of chitin to 3A5AF was also attempted in [DBU]Cl, with yields reported in Table S9. Despite affording comparable yields to aprotic IL systems, the yields remained low, highlighting that the difficulty in achieving one‐pot conversion of chitin to 3A5AF is not overcome by the use of PILs.

### Green Metrics

The overall “greenness” of this process was assessed by calculating the environmental factor (E‐factor); the ratio of mass of waste per mass of desired product. The E‐factor of a reaction process provides insight into the efficiency of the process in terms of waste generation, with the ideal value being zero.^[41]^ Table 7 compares the E‐factor of both the spectroscopic yield and isolated yields for our PIL method with other literature methods. Details of these calculations can be found in the Supporting Information.

**Table 7:** Calculated E‐factors for the synthesis of 3A5AF using different methods. The NAG loading was relative to the solvent used, and the additive loadings were relative to NAG.

Solvent	Additives	E‐factor (spectroscopic)	E‐factor (isolated)
[DBU]Cl (5 g NAG)	B(OH)_3_	40.5	1260 507^[a]^
[DBU]Cl (0.1 g NAG)	B(OH)_3_	37.1	10900 3650^[a]^
[TPA]Cl	B(OH)_3_	38.0	12200 4110^[a]^
[TBA]Cl	B(OH)_3_	33.9	11500 3850^[a]^
[C_4_C_1_im]Cl^[30]^	B(OH)_3_	20.1	1070
DMF^[22]^	AlCl_3_.6H_2_O	99.8	1250
DMF^[23]^	NH_4_Cl, LiCl	205	938 585^[a]^
GVL^[24]^	NH_4_SCN, HCl	77.3	1430
Tetrapropylammonium chloride, TsOH^[32]^	B(OH)_3_, NaCl	23.4	4740, 1340^[a]^
DMA^[14]^	[Gly]Cl,^[b]^ CaCl_2_	243	N/A
DMA^[15]^	[TEtA]Cl	210	N/A
NMP^[18]^	[PDCMPi]Cl^[c]^	314	N/A
DMA^[16]^	B(OH)_3_, CaCl_2_.2H_2_O, ChCl, CA^[d]^	151	N/A
NMP^[19]^	MgCl_2_.6H_2_O	339	N/A
DMA^[17]^	ChCl^[d]^, Glycerol, B(OH)_3_	758	N/A
NMP^[20]^	[CMPy]Cl^[e]^, B_2_O_3_, CaCl_2_	207	N/A
ChCl: PEG200: B(OH)_3_ (2 : 2 : 1)^[6]^	N/A	223	N/A
DMA^[13]^	B(OH)_3_, NaCl	44.3	N/A
NMP^[21]^	SrCl_2_, B(OH)_3_	269	N/A
Proline: glycerol: lactic acid (1 : 5 : 1)^[33]^	N/A	79.1	N/A
DMA^[43]^	Al(4.0)‐Mont^[f]^, NaCl	200	N/A

[a] Excluding water, [b] Glycine hydrochloride ([Gly]Cl), [c] 4,4′‐(propane‐1,3‐diyl)bis(1‐(carboxymethyl)piperidin‐1‐ium) chloride ([PDCMPi]Cl), [d] Choline chloride (ChCl) and citric acid (CA), [e] 1‐carboxymethyl pyridinium chloride ([CMPy]Cl), [f] Al‐exchanged montmorillonite.

Comparing first the E‐factors for the spectroscopic yields, it is apparent that the IL methods reported here and using [C_4_C_1_im]Cl and tetrapropylammonium chloride lead to E‐factors considerably less than most using organic solvents (<40). The one exception was the DMA procedure published by Omari et al. which has an E‐factor of 44.3; however, this requires temperatures of 220 °C.^[13]^ Even these leading E‐factors are large, although these values are predominantly due to solvent use and don't include the potential for recycling the solvent which would lower these values. For example, a 90 % solvent recovery would reduce the larger scale [DBU]Cl E‐factor to 8.2. The lower E‐factors for the IL and DES systems are likely due to the higher solubility of NAG and 3A5AF in these systems, enabling a reduction in the mass of solvent used.

The E‐factor for the isolated 3A5AF was based on reported solvent volumes where available or the assumption of 100 g of stationary phase and 1 L of solvent per 1 g of product.^[42]^ Water is sometimes excluded from E‐factor calculations so both water inclusive and exclusive values are indicated in Table 7.^[41]^ The E‐factor on an isolated yield basis is notably higher by more than an order of magnitude with respect to the spectroscopic yield. For the small‐scale reactions here, very large E‐factors>10000 are observed owing to the relatively small quantities of isolated product obtained from the extraction procedure. The more efficient extraction of the larger scale reaction permits a reduction in E‐factor by an order of magnitude, with the water‐exclusive E‐factor of 507 being lower than all other isolation methods, including from [C_4_C_1_im]Cl.

As noted in the introduction, [C_4_C_1_im]Cl requires 22 synthetic steps to prepare from raw materials. In comparison, [DBU]Cl requires 18, while [TPA]Cl and [TBA]Cl require only 12 (see Figures S3–S5 for synthesis trees). This illustrates that the PILs reported here, particularly with further optimisation, have the potential to provide a greener alternative to existing IL and organic solvent based methods.

## Conclusions

This study has demonstrated the conversion of NAG into 3A5AF within chloride‐based PILs. Optimisation of several reaction parameters in [DBU]Cl revealed 3A5AF can be produced in moderate yields (42.5 %) with the addition of B(OH)_3_ only, with the PILs [TPA]Cl and [TBA]Cl achieving higher yields of 51.5 % and 57.0 %. 3A5AF could be isolated in high purity by solvent extraction without the use of column chromatography from all 3 PILs investigated, with [TPA]Cl and [DBU]Cl enabling the use of a more environmentally benign EtOAc extraction. Both [TPA]Cl and [DBU]Cl could be recycled for up to 3 cycles at small (0.1 g NAG) scales, with the [DBU]Cl system shown to be effective at multigram scales (5 g NAG). These systems were shown to have lower E‐factors than 3A5AF formation from organic solvents, with lower embedded cost and waste generation due to their simpler chemical syntheses compared to aprotic ILs. This illustrates that PILs have the potential to provide more efficient access to this potentially valuable compound.

## Experimental Section

### Materials and Equipment

The following chemicals were purchased from commercial suppliers in the highest purity available and used as received: *N*‐acetyl‐D‐glucosamine, boric acid, hydrochloric acid, sulfuric acid, ethanolamine, tripropylamine, tributylamine, 1,8‐diazabicyclo[5.4.0]undec‐7‐ene, Aliquat 336, triethanolamine hydrochloride and dimethylamine hydrochloride. 1,5‐diazobicyclo[4.3.0]non‐5‐ene was distilled under reduced pressure prior to its use. All PILs were synthesised by a stoichiometric reaction between hydrochloric acid and the corresponding amine, as detailed in the Supporting Information and were dried overnight *in vacuo* at 60 °C before use. Chitin was ground in dry mode with a planetary ball mill at a rotational speed of 250 rpm and milling time of 1 h to reduce the particle size, mild grinding conditions were used to avoid hydrolysis and degradation of the chitin.

NMR spectra were recorded on a Bruker Avance 400 MHz spectrometer. High‐Performance Liquid Chromatography (HPLC) analysis was performed using Thermo Scientific UltiMate 3000 with an Aminex 87H column and a UV‐Vis detector. The mobile phase consisted of 40 % acetonitrile and 60 % 5 mM sulfuric acid water at a flow rate of 0.6 mL min^−1^ at 55 °C. The 3A5AF standard was prepared according to literature methods;^[13]^ a calibration curve was then plotted and employed for the quantification of 3A5AF (Figures S1 and S2).

### General Procedure for NAG Conversion in ILs

In a typical reaction, NAG and additives were added to 1 g of IL in a glass vial (4 mL or 20 mL). The reaction mixture was heated in a aluminium heating block at the selected temperature. For kinetic analysis, 50 μL aliquots were taken at regular intervals, then analysed by HPLC. All reactions followed the general procedure while varying some parameters, including IL solvent, substrate loading, type and amount of additive and temperature (Table S1). The reaction time varied between 60–180 min for all conditions employed.

### Product Quantification

Accurately weighed sample aliquots (50 μL) were dissolved in 2 mL of the HPLC eluent (2 : 3 volume ratio acetonitrile: 5 mM aqueous H_2_SO_4_). This solution was filtered by a PTFE syringe filter (0.2 μm pore size) before analysis by HPLC. The quantification of 3A5AF and NAG was conducted using calibration plots where the HPLC peak area was converted to weight percent (wt %) in solution. The calibration plots were used to determine the amount of product or starting material (wt %) in the HPLC sample, then a dilution factor was used to transform this into the absolute yield in the reaction solution. Reactions were performed in duplicate or triplicate and the average 3A5AF yields and NAG conversion were calculated. The experimental error was estimated as the standard deviation of replicate experiments.

### 3A5AF Extraction

For the small scale (1 g IL) reactions, following completion of the reaction, the reaction mixture was immediately dissolved in water (10 mL), which was then washed with ethyl acetate (100 mL). For the [DBU]Cl system, the ethyl acetate layer was washed with 0.1 M NaOH (100 mL) then pure water (100 mL). For the [TPA]Cl and [TBA]Cl systems, the ethyl acetate was washed with 0.1 M HCl instead of 0.1 M NaOH. Finally, the ethyl acetate was removed by rotary evaporator to yield 3A5AF as a pale brown solid.

For the larger scale reaction (50 g IL), solvent amounts were as follows: 250 mL water for reaction mixture dissolution, 500 mL ethyl acetate, 250 mL 0.1 M NaOH and 250 mL water for purification.

### Recycling Procedure

The recyclability of [DBU]Cl and [TPA]Cl were examined using the following general procedure. NAG (0.10 g, 0.45 mmol or 5.0 g, 23 mmol) and B(OH)_3_ (0.056 g, 0.90 mmol or 0.28 g, 45 mmol) were added to [DBU]Cl or [TPA]Cl (1.0 g or 50 g) and heated at 150 °C. The 3A5AF formed was extracted using the method outlined above. Following extraction, water and excess EtOAc were removed by rotary evaporator, then the reaction solution was dried at 60 °C in vacuo for 24 h. The recovered IL was then reused by adding either the same mass of NAG and B(OH)_3_ again or additional NAG only.

## Conflict of Interests

The authors declare no conflict of interest.

## Supporting information

As a service to our authors and readers, this journal provides supporting information supplied by the authors. Such materials are peer reviewed and may be re‐organized for online delivery, but are not copy‐edited or typeset. Technical support issues arising from supporting information (other than missing files) should be addressed to the authors.

Supporting Information

## Data Availability

The data that support the findings of this study are available in the supplementary material of this article.
