# Supplementary material for: Efficient Production and Isolation of 3‐Acetamido‐5‐Acetylfuran from N‐Acetyl‐D‐Glucosamine within Protic Ionic Liquids
Source: ChemistryOpen. 2025 Feb 25;14(9):e202500094. doi: 10.1002/open.202500094 (PMC12409840; doi:10.1002/open.202500094)
Supplement: Supplementary file 1 — Supporting Information [file OPEN-14-e202500094-s001.pdf]

ChemistryOpen

Supporting Information

Efficient Production and Isolation of 3-Acetamido-5-Acetylfuran from N-Acetyl-D-Glucosamine within Protic Ionic Liquids

Emma L. Matthewman, Jonathan Sperry, and Cameron C. Weber*

ChemistryOpen

Supporting Information

Efficient Production and Isolation of 3-Acetamido-5-acetylfuran from N-Acetyl-D-glucosamine within Protic Ionic Liquids

Emma L. Matthewman, Jonathan Sperry and Cameron C. Weber

Ionic Liquid Synthesis

Synthesis of 1,8-Diazobicyclo[5.4.0]undec-7-ene Chloride ([DBU]Cl)

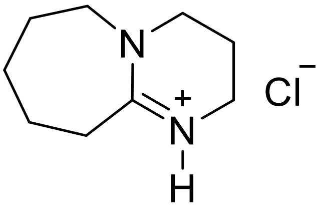

Varying the procedure outlined by Liu et al.,[1] hydrochloric acid (37%, 87.1 mL, 1.05 mol) was added dropwise to 1,8-diazobicyclo[5.4.0]undec-7-ene (161 g, 1.05 mol) in a two-neck flask cooled in an ice bath. After the mixture was stirred at 50 °C for 24 h, a pale-yellow viscous liquid was obtained. The resultant solution was then dried *in vacuo* at 80 ℃ for 24 h. 1,8-diazobicyclo[5.4.0]undec-7-ene chloride ([DBU]Cl) was afforded as a white crystalline solid (98.9%, 196 g, 1.04 mol).

^{1}H NMR δ (ppm) (400 MHz, DMSO-d_6): 10.00 (s, 1H), 3.58 (m, 1H), 3.49 (t, J = 5.8 Hz, 1H), 3.25 (t, J = 5.7 Hz, 1H) 2.83-2.62 (m, 1H), 1.98-1.93 (m, 1H), 1.78-1.53 (m, 4H)

^{13}C{^{1}H} NMR δ (ppm) (101 MHz, DMSO-d_6): 165.36, 53.30, 47.86, 37.42, 31.30, 28.26, 25.96, 23.37, 18.89

Synthesis of 1,5-Diazobicyclo[4.3.0]non-5-ene Chloride ([DBN]Cl)

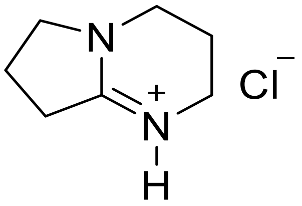

Using the general method of Liu et al.,[1] hydrochloric acid (37%, 1.71 mL, 0.0205 mol) was added dropwise to 1,5-diazobicyclo[4.3.0]non-5-ene (2.54 g, 0.0205 mol) in a round bottom flask cooled in an ice bath. The resultant solution was stirred at 50 °C for 5 h, then dried in vacuo at 80 °C for 2 h to obtain 1,5-diazobicyclo[4.3.0]non-5-ene chloride ([DBN]Cl) as a viscous pale-yellow liquid (3.14 g, 0.195 mol, 95.1%).

^{1}H NMR δ (ppm) (400 MHz, DMSO-d_6): 8.19 (s, 1H), 3.49-3.18 (m, 6H), 2.83 (t, J = 8.0 Hz, 2H), 1.99-1.88 (m, 4H)

$^{13}C\{^{1}H\}$ NMR δ (ppm) (101 MHz, DMSO-d_6): 163.82, 52.99, 46.34, 41.95, 37.51, 24.89, 18.28

Synthesis of Tripropylammonium Chloride ([TPA]Cl)

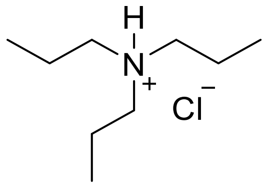

According to the general method of Liu et al.,[1] hydrochloric acid (37%, 6.64 mL, 0.0797 mol) was added dropwise to tripropylamine (11.4 g, 0.0797 mol) in a round bottom flask cooled in an ice bath. The resultant solution was stirred at room temperature for 6 h, then dried in vacuo at 50 °C for 24 h to afford tripropylammonium chloride ([TPA]Cl) as a white crystalline solid (13.9 g, 0.0771 mol, 96.7%).

^{1}H NMR δ (ppm) (400 MHz, DMSO-d_6): 10.13 (s, 1H), 3.06 – 2.79 (m, 6H), 1.85-1.45 (m, 6H), 0.92 (t, J = 7.4 Hz, 9H).

$^{13}C\{^{1}H\}$ NMR δ (ppm) (101 MHz, DMSO-d_6): 53.27, 16.43, 10.87

Synthesis of Tributylammonium Chloride ([TBA]Cl)

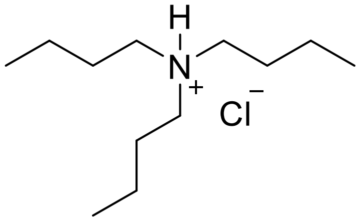

Using the general method of Liu et al.,[1] hydrochloric acid (37%, 4.73 mL, 0.0567 mol) was added dropwise to tributylamine (10.5 g, 0.0567 mol) in a round bottom flask cooled in an ice bath. The resultant solution was stirred at room temperature for 5 h, then dried in vacuo at 50 °C for 24 h to afford tributylammonium chloride ([TBA]Cl) as a white crystalline solid (12.3 g, 0.0555 mol, 97.8%).

^{1}H NMR δ (ppm) (400 MHz, DMSO-*d*$_6$): 10.70 (s, 1H), 3.12-2.75 (m, 6H), 1.79-1.51 (m, 6H), 1.46-1.19 (m, 6H), 0.92 (t, *J* = 7.4 Hz, 9H).

^{13}C{^{1}H} NMR δ (ppm) (101 MHz, DMSO-*d*$_6$): 51.40, 24.76, 19.48, 13.52

Synthesis of 1-Methylimidazolium Chloride ([C$_1$im]Cl)

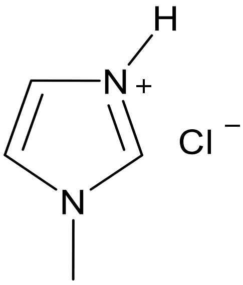

According to the method of Ohno and Yoshikawa,[2] hydrochloric acid (37%, 3.20 mL, 0.0384 mol) was added dropwise to 1-methylimidazole (3.15 g, 0.0384 mol) in a round bottom flask cooled in an ice bath. The mixture was stirred at room temperature for 12 h, then dried in vacuo at 60 °C for 24 h to yield 1-methylimidazolium chloride ([C$_1$im]Cl) as a white crystalline solid (4.42 g, 0.0373 mol, 97.1%).

^{1}H NMR δ (ppm) (400 MHz, DMSO-*d*$_6$): 9.16 (s, 1H), 7.72 (t, *J* = 1.6 Hz, 1H), 7.66 (t, *J* = 1.6 Hz, 1H), 3.88 (s, 3H)

^{13}C{^{1}H} NMR δ (ppm) (101 MHz, DMSO-*d*$_6$): 135.59, 123.08, 119.53, 35.33

Synthesis of Ethanolammonium Chloride ([EtA]Cl)

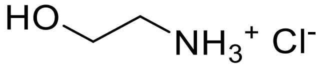

Using the method of Sun et al.,[3] hydrochloric acid (37%, 3.30 mL, 0.0396 mol) was added dropwise to ethanolamine (2.42 g, 0.0396 mol) in a round bottom flask cooled in an ice bath. The resultant mixture was stirred at room temperature 12 h, then dried in vacuo at 60 °C for 24 h to afford ethanolammonium chloride ([EtA]Cl) as a white crystalline solid (3.78 g, 0.0388 mol, 98.0%).

^{1}H NMR δ (ppm) (400 MHz, DMSO-*d*$_6$): 7.89 (s, 3H), 3.66 – 3.55 (m, 2H), 2.84 (m, 2H)

^{13}C{^{1}H} NMR δ (ppm) (101 MHz, DMSO-*d*$_6$): 57.42, 41.20

Reaction Procedures for the Conversion of NAG to 3A5AF in [DBU]Cl

In a typical reaction, NAG and additives were added to 1 g of [DBU]Cl in a 4 mL glass vial. The reaction mixture was heated in an aluminium heating block at the selected temperature. For kinetic analysis, 50 µL aliquots were taken at regular intervals, then analysed by HPLC. All reactions followed this general conversion procedure while varying some parameters such as the substrate loading, type and amount of additive and temperature (Table S1). The reactions performed using all other chloride-based PILs were conducted using the conditions outlined in Entry 2 and 3. The reaction time varied between 60-180 min for all conditions employed.

Table S1 A summary of the reaction conditions employed for the conversion of NAG to 3A5AF in [DBU]Cl

Entry	NAG (mg)	Temperature (℃)	$B(OH)_3$ (mg)	HCl (µL)	NaCl (mg)
1	50	120	28.0	-	-
2	50	150	28.0	-	-
3	50	180	28.0	-	-
4	50	150	14.0	-	-
5	50	150	55.9	-	-
6	50	150	28.0	18.8	-
7	50	150	28.0	-	26.4
8	100	150	111.8	-	-
9	250	150	279.5	-	-

Reaction Procedures for the Conversion of Chitin to 3A5AF in [DBU]Cl

In a typical reaction, ball-milled chitin and additives were added to 1 g of IL in a 4 mL glass vial. The reaction mixture was heated in an aluminium heating block at the selected temperature. For kinetic analysis, 50 μL aliquots were taken at regular intervals, then analysed by HPLC. All reactions followed this general conversion procedure while varying some parameters such as the type and amount of additive and temperature (Table S2).

Table S2 A summary of the reaction conditions employed for the conversion of chitin to 3A5AF in [DBU]Cl

Entry	Chitin (mg)	Temperature (℃)	$B(OH)_3$ (mg)	HCl (μL)
1	50	150	60.9	20.5
2	50	180	60.9	20.5
3	50	150	30.5	20.5
4	50	180	30.5	20.5
5	50	180	30.5	-

HPLC Calibration Curves

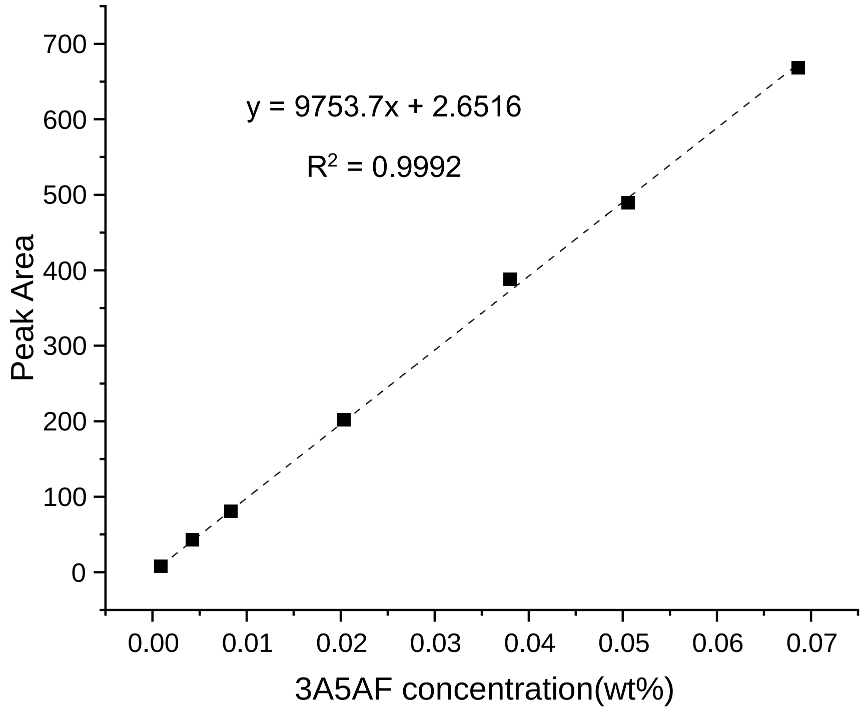

Figure S1. HPLC calibration plot for 3A5AF quantification showing integrated peak area against 3A5AF concentration.

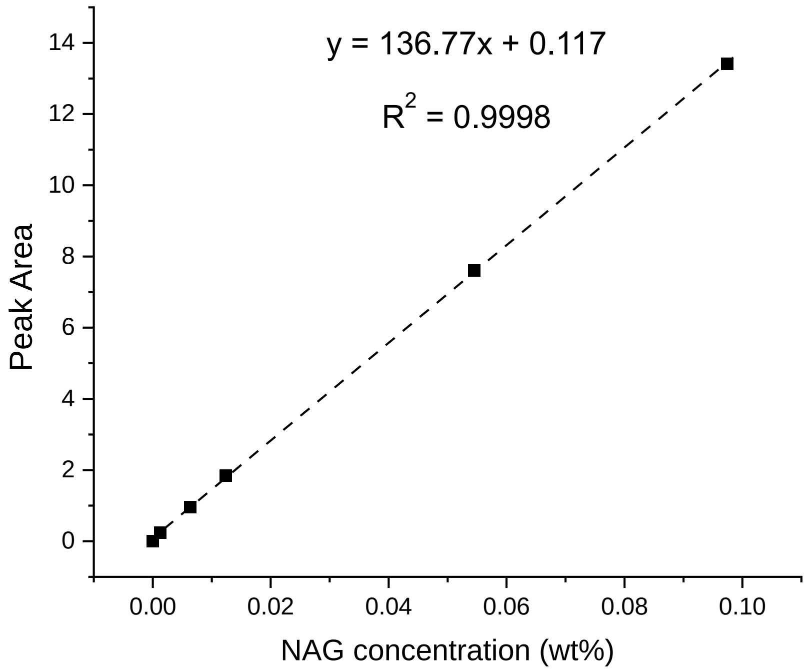

Figure S2. HPLC calibration plot for NAG quantification showing integrated peak area against NAG concentration

Synthesis Trees for PILs

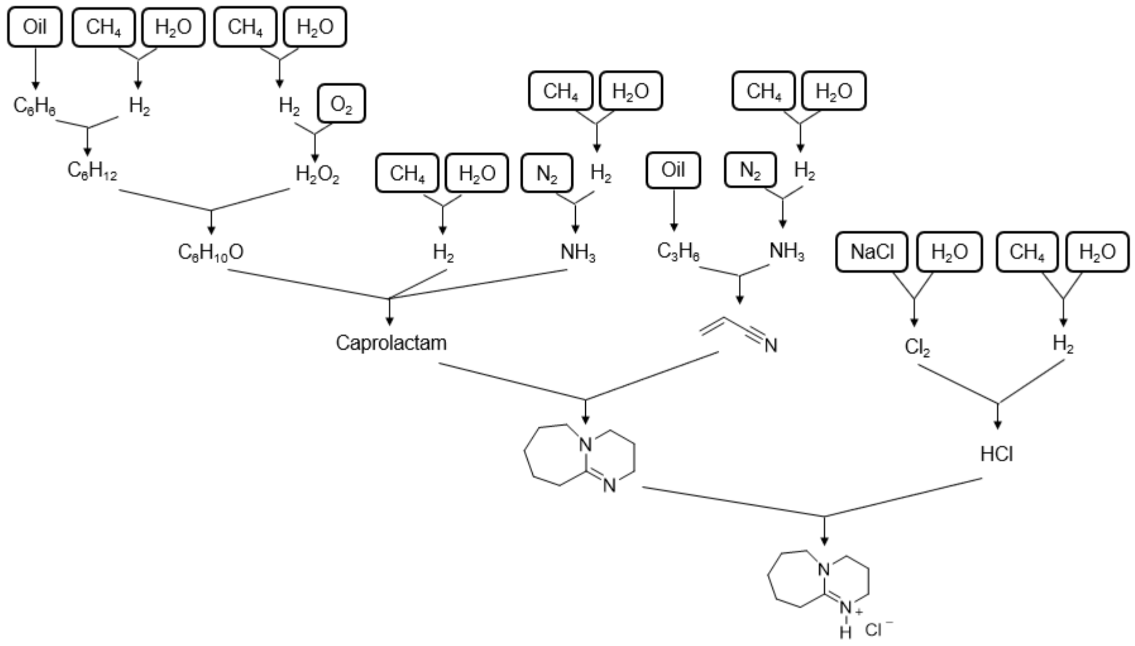

Figure S3. Synthesis tree for [DBU]Cl.

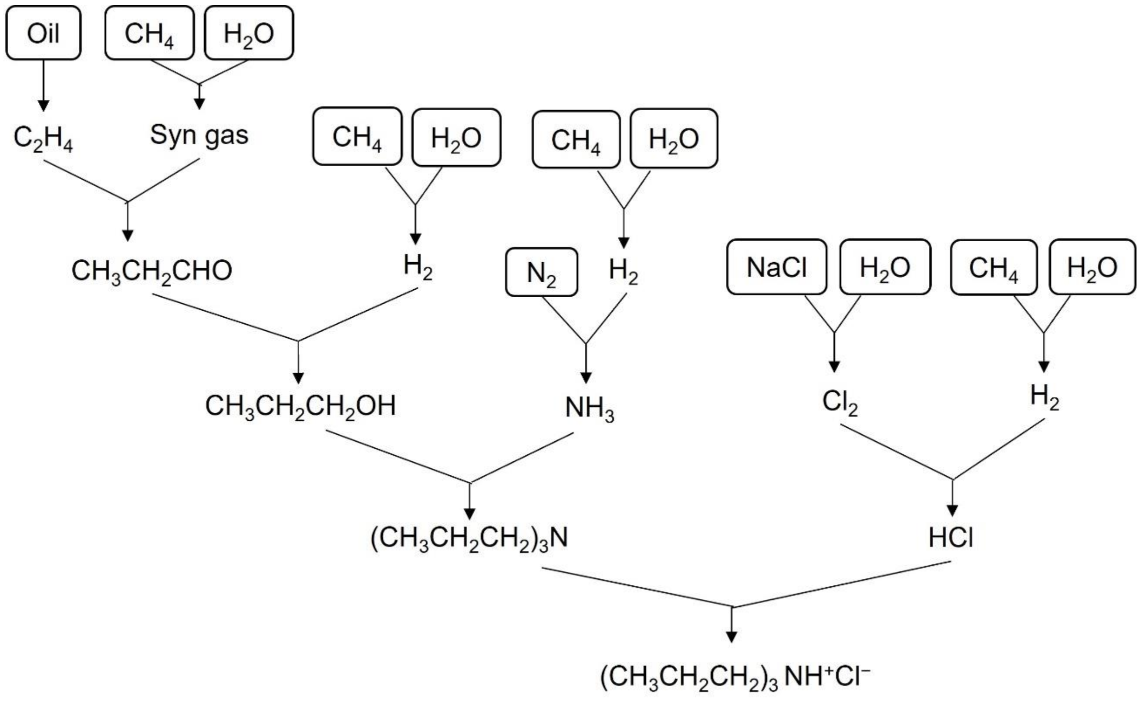

Figure S4. Synthesis tree for [TPA]Cl.

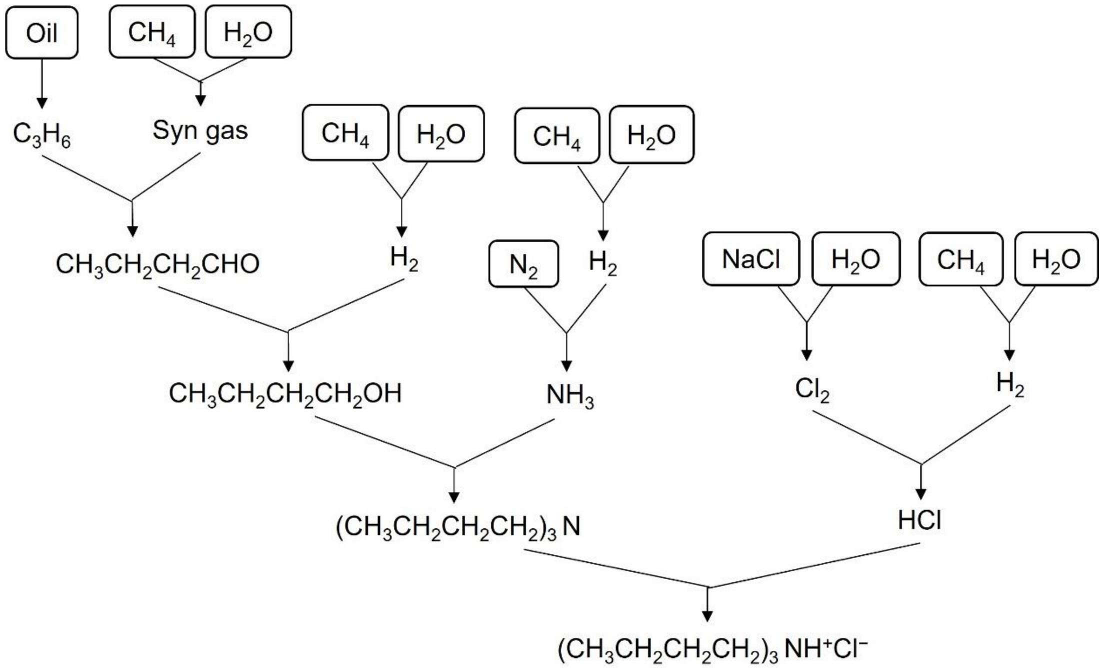

Figure S5. Synthesis tree for [TBA]Cl.

NMR Spectra for [EtA]Cl with $B(OH)_3$

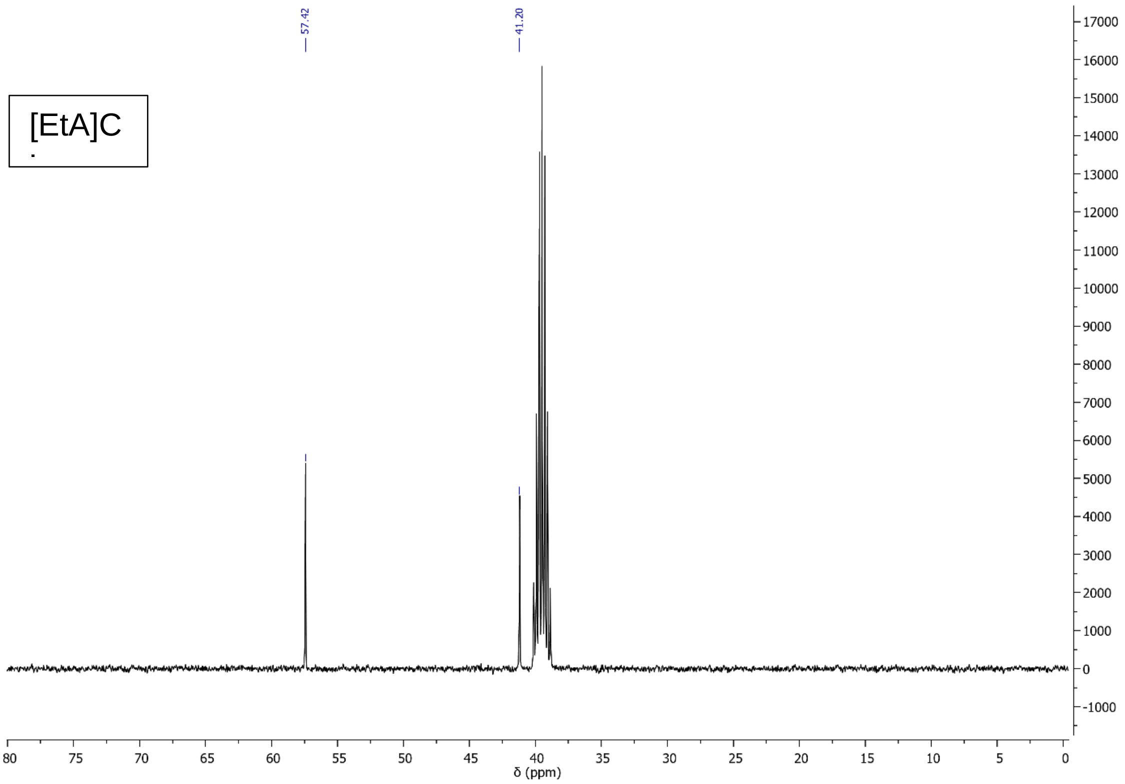

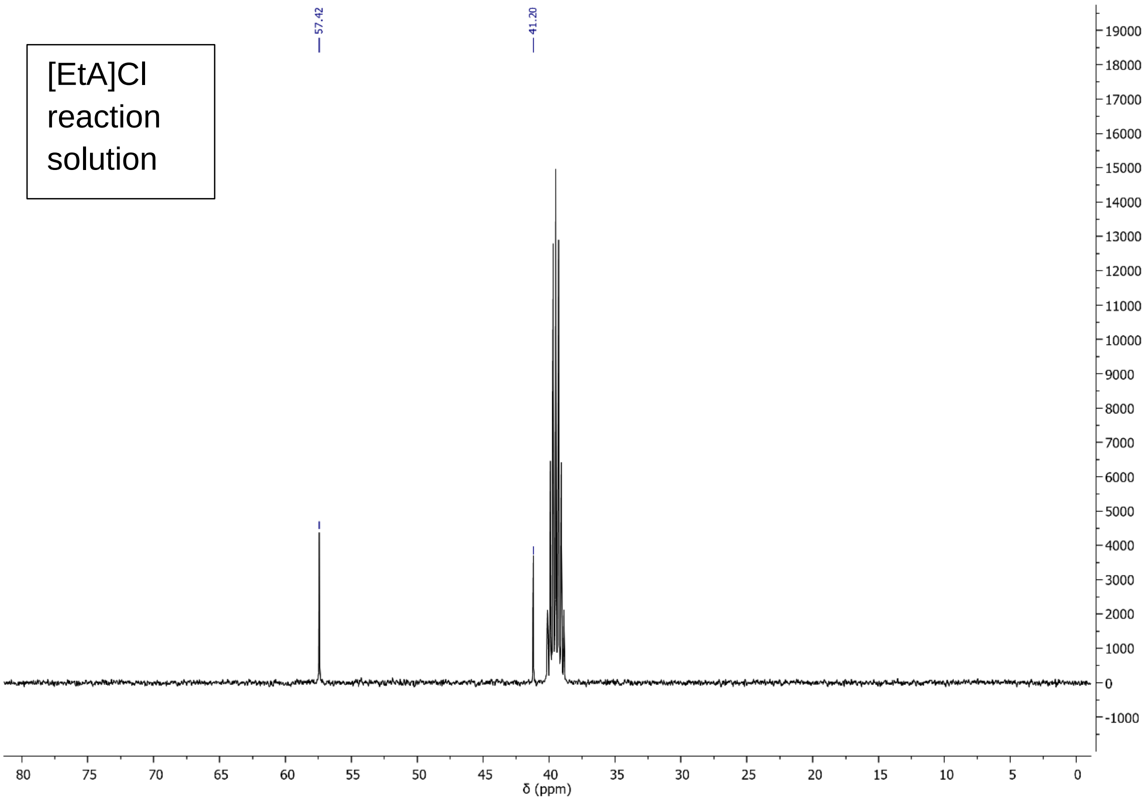

Figure S6. ^{13}C NMR spectra of [EtA]Cl (top) an [EtA]Cl reaction solution (bottom, 1 g [EtA]Cl, 5 wt% NAG relative to the IL, 200 mol% $B(OH)_3$ relative to NAG, 150 °C)

NMR Spectra of [DBN]Cl made from purified and unpurified DBN

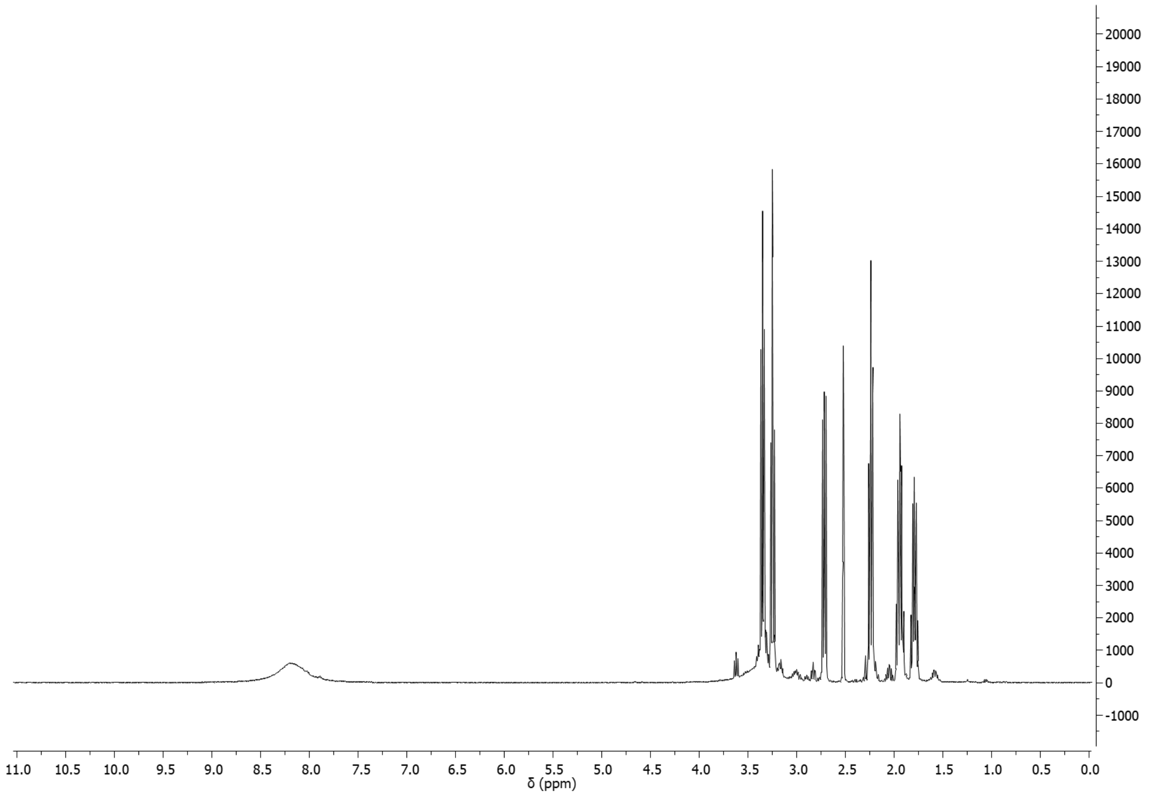

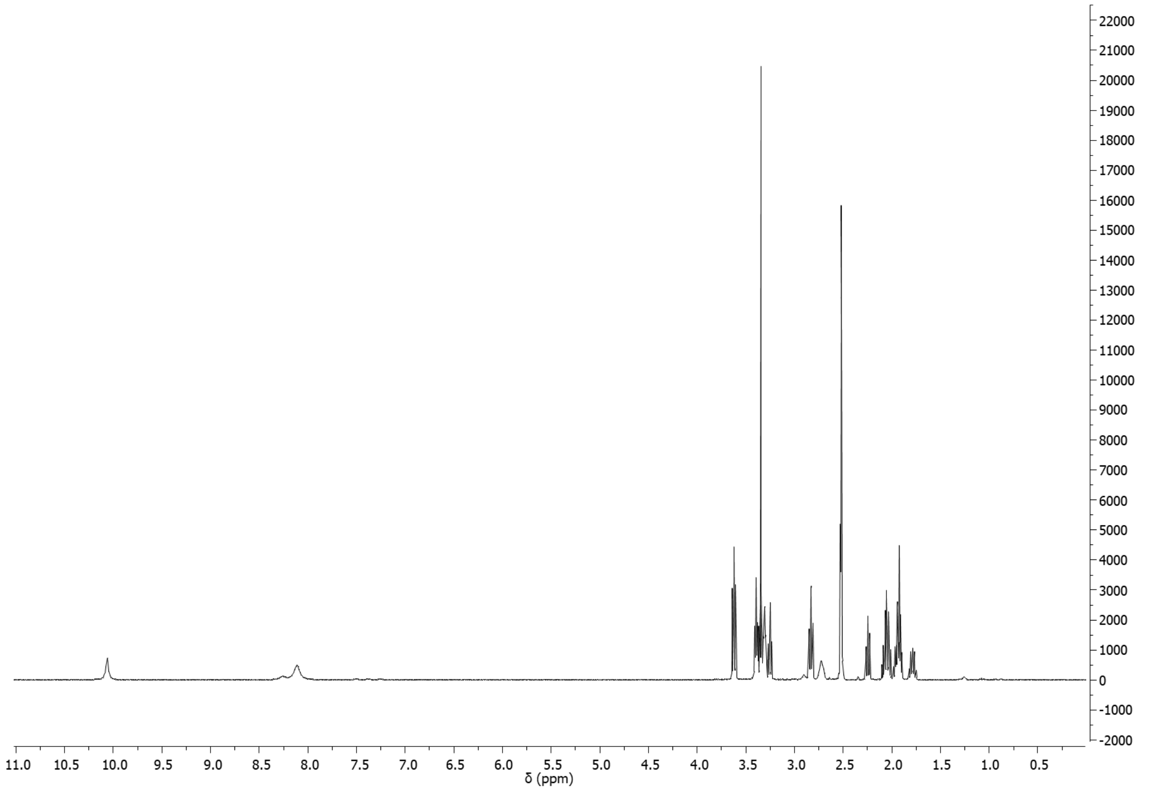

Figure S7. ^{1}H NMR spectra for [DBN]Cl made from unpurified DBN (top) and remade using distilled DBN (bottom)

Representative Isolated 3A5AF NMR Spectrum

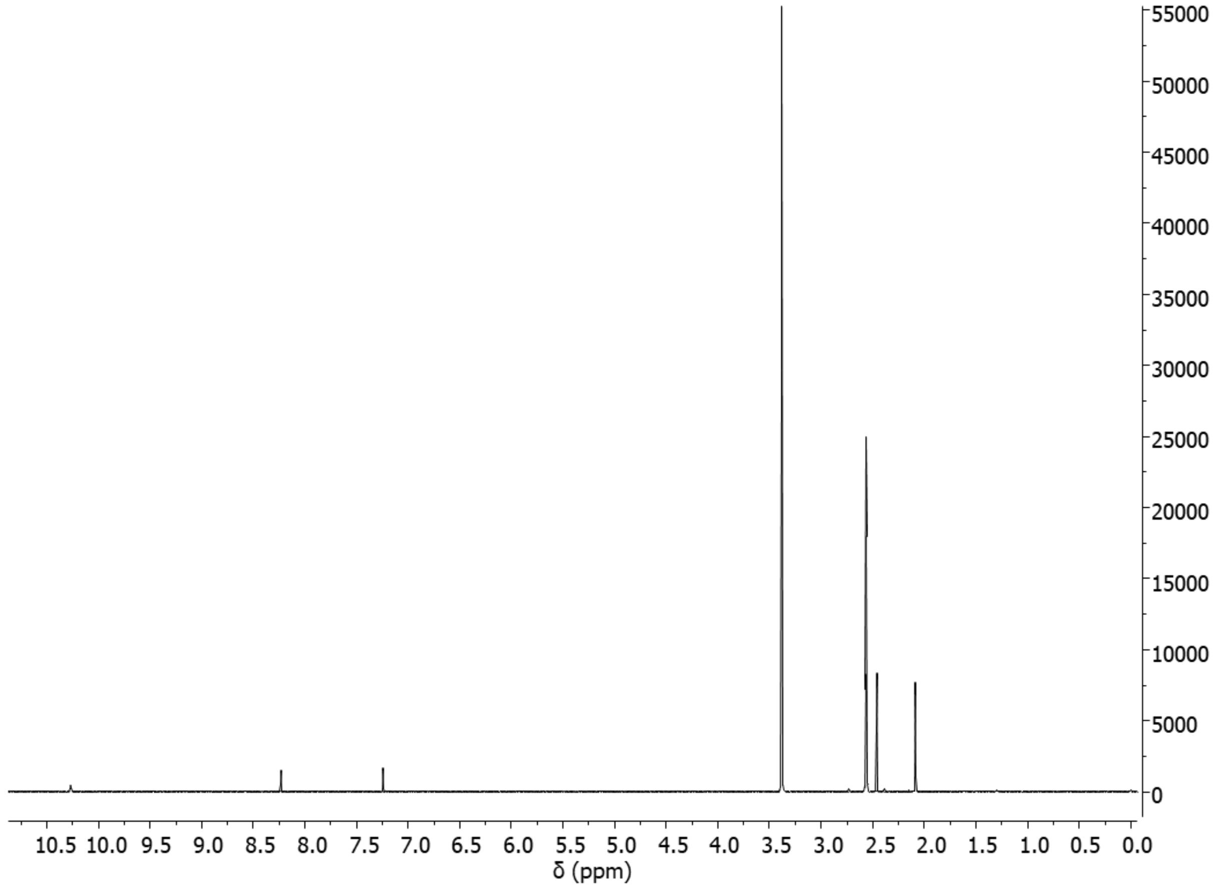

^{1}H NMR δ (ppm) (400 MHz, DMSO-d_6): 10.27 (s, 1H), 8.23 (d, J = 0.7 Hz, 1H), 7.25 (d, J = 0.8 Hz, 1H), 2.46 (s, 3H), 2.09 (s, 3H).

Figure S8. ^{1}H NMR spectrum of isolated 3A5AF

pH Measurement of PILs

pH measurements were conducted using a Mettler Toledo SevenCompact pH meter S220. pH values were obtained in aqueous solution at a concentration of 100 g L^{-1}.

Table S3. Measured pH values and 3A5AF yields obtained in each of the PILs explored

PIL	pH of PIL	3A5AF Yield (%)
A336	3.3	12.2 ± 1.7
[TPA]Cl	3.6	48.8 ± 4.6
[C_1im]Cl	4.8	18.5 ± 0.08
[TEtA]Cl	4.9	6.0 ± 1.1
[DMA]Cl	5.0	4.4 ± 0.2
[TBA]Cl	5.5	50.5 ± 1.2
[DBU]Cl	6.9	42.5 ± 1.4
[DBN]Cl	7.0	3.8 ± 3.2
[EtA]Cl	7.1	0.15 ± 0.02

Recyclability with Additional $B(OH)_3$ after Each Cycle

Table S4. HPLC and isolated yields of 3A5AF obtained in successive cycles with additional 200 mol% $B(OH)_3$ added for each cycle in [DBU]Cl

Reaction cycle	HPLC 3A5AF yield (%)	Isolated yield (%)
1	44.8	36.7
2	36.5	35.0
3	29.7	25.8

Tabular Experimental Data from Figures 2-5

Table S5. 3A5AF yields obtained using in [DBU]Cl in the absence of additives. Reaction conditions: NAG (50 mg), [DBU]Cl (1 g) at 150 °C for 2 h. Reported errors are standard deviations obtained from replicate experiments.

3A5AF yields (%)				
20 min	40 min	60 min	90 min	120 min
4.95 ± 0.75	17.1 ± 0.1	24.9 ± 0.6	32.3 ± 1.4	35.9 ± 0.3

Table S6. 3A5AF yields obtained using $B(OH)_3$ as an additive in [DBU]Cl. Reaction conditions: NAG (50 mg), [DBU]Cl (1 g) at 150 °C for 2 h. Reported errors are standard deviations obtained from replicate experiments.

$B(OH)_3$ concentration (mol%)	3A5AF yields (%)					
	10 min	20 min	40 min	60 min	90 min	120 min
400	-	3.6 ± 0.3	18.9 ± 0.8	36.3 ± 1.8	38.6 ± 0.01	-
200	4.1 ± 2.4	16.5 ± 1.7	32.1 ± 1.0	38.1 ± 0.6	38.8 ± 1.8	42.5 ± 1.4
100	10.5 ± 4.1	30.2 ± 1.7	35.2 ± 0.07	35.9 ± 1.4	34.0 ± 1.7	31.0 ± 4.6

Table S7. 3A5AF yields obtained using NaCl or HCl and $B(OH)_3$ as additives in [DBU]Cl. Reaction conditions: NAG (50 mg), $B(OH)_3$ (28 mg, 2 eq.), other additives (as shown below), [DBU]Cl (1 g) at 150 °C for 2 h. Reported errors are standard deviations obtained from replicate experiments.

NaCl loading (mol%)	HCl loading (mol%)	$B(OH)_3$ loading (mol%)	3A5AF yields (%)						
			5 min	10 min	20 min	40 min	60 min	90 min	120 min
200	-	200	-	-	4.9 ± 0.7	17.1 ± 0.1	24.8 ± 0.6	32.3 ± 1.4	35.9 ± 0.3
-	100	200	26.9 ± 2.7	45.4 ± 0.4	39.3 ± 0.9	33.2 ± 2.6	31.8 ± 2.0	-	-

Table S8. 3A5AF yields obtained at different temperatures with $B(OH)_3$ in [DBU]Cl. Reaction conditions: NAG (50 mg), $B(OH)_3$ (28 mg, 2 eq.), [DBU]Cl (1 g) at 150 °C for 2 h. Reported errors are standard deviations obtained from replicate experiments.

Temperature (°C)	$B(OH)_3$ loading (mol%)	3A5AF yield (wt%)							
		5 min	10 min	20 min	40 min	60 min	90 min	120 min	180 min
120	200	-	-	0.9 ± 0.03	6.6 ± 0.5	12.5 ± 0.7	18.0 ± 1.6	26.5 ± 2.6	33.6 ± 3.9
180	200	28.9 ± 3.2	31.5 ± 0.6	33.7 ± 4.6	25.9 ± 0.5	22.1 ± 0.8			

Results of Chitin to 3A5AF Experiments in [DBU]Cl

Table S9. Yields of 3A5AF obtained from chitin in [DBU]Cl under different reaction conditions. Reaction conditions: chitin (50 mg), [DBU]Cl (1 g), temperature and additive concentration as shown below. Reported errors are standard deviations obtained from replicate experiments

Temperature (°C)	HCl Concentration (mol%)	$B(OH)_3$ Concentration (mol%)	3A5AF Yield (%)
			120 min
150	-	200	-
150	100	200	5.2 ± 0.1
150	100	400	3.2 ± 0.3
180	100	200	4.2 ± 0.1
180	100	400	4.0 ± 0.4

E-factor Calculations

The environmental-factor (E-factor) for 3A5AF preparation was determined using Equation S1.

$$E - factor = \frac{mass\ of\ total\ waste}{mass\ of\ product} \qquad \text{Equation S1}$$

The masses used to calculate this for the E-factors reported in Table 7 are summarised in Table S10 for spectroscopic yields and Table S11 for isolated yields. The mass of reactants in Table S11 is the sum of the mass of NAG, solvent and additives from Table S10. Silica and solvent volumes in Table S11 are either reported volumes or assumptions made on the basis of 100 g of stationary phase and 1 L of solvent per 1 g of product isolated by column chromatography.

Table S10. Inputs used to calculate E-factors for spectroscopic yields.

Solvent	Additive	Yield (%)	NAG (g)	Solvent (g)	Additives (g)	Product (g)	E-factor
[DBU]Cl	$B(OH)_3$	36.9	5	50	2.8	1.39	40.6
[DBU]Cl	$B(OH)_3$	44.8	0.1	1	0.19	0.0339	37.1
[TPA]Cl	$B(OH)_3$	43.8	0.1	1	0.19	0.0331	38.0
[TBA]Cl	$B(OH)_3$	48.9	0.1	1	0.19	0.0370	33.9
$[C_4C_1im]Cl$[4]	$B(OH)_3$	60	0.1	0.75	0.053	0.0427	20.1
DMF[5]	$AlCl_3.6H_2O$	29	4.72	94.4	5.15	1.03	99.8
DMF[6]	NH_4Cl, LiCl	41.9	3	188.8	3.72	0.949	205
GVL[7]	NH_4SCN, HCl	75.3	0.1	4.2	0.154	0.0569	77.3
DMA[8]	[Gly]Cl, $CaCl_2$	52.6	0.1	9.4	0.2	0.0398	243
DMA[9]	[TEtA]Cl	62.8	0.1	9.4	0.5	0.0475	210
NMP[10]	[PDCMPi]Cl	43.9	0.1	10.3	0.0542	0.0332	314
DMA[11]	$B(OH)_3$, $CaCl_2.2H_2O$, ChCl, CA	47.1	0.094	4.7	0.278	0.0335	151
NMP[12]	$MgCl_2.6H_2O$	41.6	0.1	10.3	0.276	0.0314	339
DMA[13]	ChCl, Glycerol, $B(OH)_3$	37.7	0.0221	4.7	0.0587	0.0063	758
NMP[14]	[CMPy]Cl, B_2O_3, $CaCl_2$	68.2	0.1	10.3	0.3	0.0515	207
ChCl: PEG200: $B(OH)_3$ (2:2:1)[15]	N/A	18.3	0.1	3	0	0.0138	223
DMA[16]	$B(OH)_3$, NaCl	57.7	0.236	4.23	0.190	0.103	44.3
NMP[17]	$SrCl_2$, $B(OH)_3$	51.9	0.1	10.3	0.171	0.0392	269
Proline: glycerol: lactic acid (1:5:1)[18]	N/A	39	0.221	5	0	0.0652	79.1
Tetrapropylammonium chloride, TsOH[19]	$B(OH)_3$, NaCl	70	0.25	2.64	0.34	0.1322	23.4
DMA[20]	Al(4.0)-Mont, NaCl	14	1.5	28.2	2.12	0.1587	200

Table S11. Inputs used to calculate E-factors for isolated yields.

Solvent	Additive	Yield (%)	Reactants (g)	Silica (g)	Solvent (g)	Product (g)	E-factor
[DBU]Cl	$B(OH)_3$	26.6	57.8	0	1201[a]	1.00	1260<br>507[b]
[DBU]Cl	$B(OH)_3$	36.7	1.29	0	300[c]	0.0277	10900<br>3650[b]
[TPA]Cl	$B(OH)_3$	32.6	1.29	0	300[c]	0.0246	12200<br>4110[b]
[TBA]Cl	$B(OH)_3$	34.8	1.29	0	300[c]	0.0263	11500<br>3850[b]
$[C_4C_1im]Cl$[4]	$B(OH)_3$	57	0.903	10	32.5	0.0406	1070
DMF[5]	$AlCl_3.6H_2O$	18	104	70	700	0.7	1250
DMF[6]	NH_4Cl, LiCl	45	196	0	1400[d]	1.7	938<br>585[b]
GVL[7]	NH_4SCN,<br>HCl	54	4.45	0.1[e]	84[f]	0.0408	2170<br>1430[b]
Tetrapropylammonium chloride, TsOH[19]	$B(OH)_3$,<br>NaCl	68	12.92	0	2426.6[g]	0.5145	4740<br>1340[b]

[a] Includes 750 g water, [b] Excludes water, [c] Includes 200 g water, [d] Includes 200 g water, [e] Activated carbon as sorbent rather than silica, [f] Includes 30 g water, [g] Includes 1750 g water.

Attempted Quantification of Glucosamine

Glu is a potential by-product formed from the hydrolysis of the N-acetyl group of NAG. Since Glu was protonated under the HPLC conditions employed, it eluted with the solvent front, making it unable to be identified and quantified using the existing HPLC method. Quantification of Glu was attempted by derivatisation with FmocCl, to protect the C-5 amine group prior to analysis by HPLC. FmocCl was selected as it is widely employed for the derivatisation and analysis of primary and secondary amines.

This approach generated satisfactory calibration curves for Glu (Figure S9). However, when this approach was used for the quantification of Glu in a reaction mixture featuring chitin as a substrate, highly inconsistent results were observed even for reaction performed under identical reaction conditions (Table S12). The inconsistency in yields obtained was first thought to be due to unreacted chitin inhibiting the FmocCl protection of Glu. To overcome this, Glu

quantification was attempted again by filtering each sample through a syringe filter prior to the addition of FmocCl to remove any insoluble, unreacted substrate. As shown in Table S12, this did not improve the reproducibility of the results. Since accurate data was unable to obtained after several trials, the quantification of Glu was not pursued further.

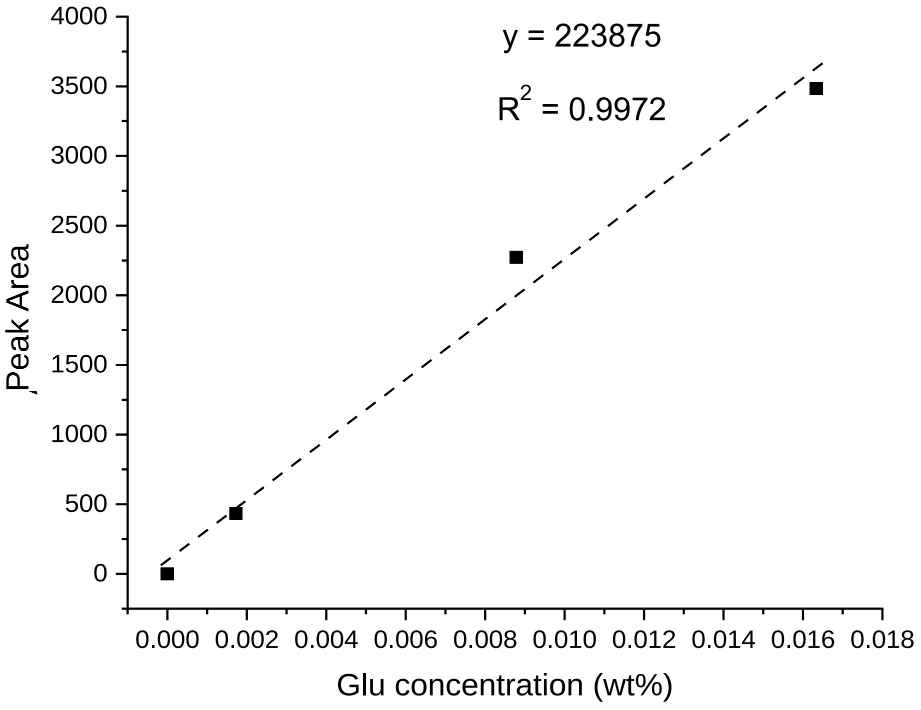

Figure S9. HPLC calibration curve for Glu following FmocCl protection.

Table S12. Glu yields determined after FmocCl protection by HPLC from a reaction mixture consisting of chitin in [C_4C_1im]Cl. Filtered samples were obtained from the same solution and but passed through a syringe filter to remove unreacted chitin prior to FmocCl protection.

	**Glu yield (wt%)**			
**Time (min)**	**Unfiltered samples**		**Filtered samples**	
10	11.0	5.6	13.7	-
20	13.8	5.8	12.4	4.5
40	15.1	6.4	13.1	1.7
60	16.5	6.4	14.7	1.7

References

[1] M. Liu, J. Guo, Y. Gu, J. Gao, F. Liu, *Polymer Degradation and Stability* **2018**, *157*, 9-14.

[2] H. Ohno, M. Yoshizawa, *Solid State Ionics* **2002**, *154-155*, 303-309.

[3] J. Sun, N. V. S. N. M. Konda, R. Parthasarathi, T. Dutta, M. Valiev, F. Xu, B. A. Simmons, S. Singh, *Green Chem.* **2017**, *19*, 3152-3163.

[4] M. W. Drover, K. W. Omari, J. N. Murphy, F. M. Kerton, *RSC Adv.* **2012**, *2*, 4642-4644.

[5] D. Padovan, H. Kobayashi, A. Fukuoka, *ChemSusChem* **2020**, *13*, 3594-3598.

[6] C. Wang, C. Wu, A. Zhang, K. Chen, F. Cao, P. Ouyang, *ChemistrySelect* **2022**, *7*, e202104574.

[7] X. Ji, J. Kou, G. Gözaydın, X. Chen, *Appl. Catal. B: Environ.* **2024**, *342*, 123379.

[8] J. Wang, H. Zang, S. Jiao, K. Wang, Z. Shang, H. Li, J. Lou, *Sci. Total Environ.* **2020**, *710*, 136293.

[9] H. Zang, H. Li, S. Jiao, J. Lou, Y. Du, N. Huang, *ChemistrySelect* **2021**, *6*, 3848-3857.

[10] H. Zang, Y. Feng, J. Lou, K. Wang, C. Wu, Z. Liu, X. Zhu, *J. Mol. Liq.* **2022**, *366*, 120281.

[11] C. Wu, C. Wang, A. Zhang, K. Chen, F. Cao, P. Ouyang, *React. Chem. Eng.* **2022**, *7*, 1742-1749.

[12] H. Zang, Y. Feng, M. Zhang, K. Wang, Y. Du, Y. Lv, Z. Qin, Y. Xiao, *Carbohydr. Res.* **2022**, *522*, 108679.

[13] J. Zhao, C. M. Pedersen, H. Chang, X. Hou, Y. Wang, Y. Qiao, *iScience* **2023**, *26*, 106980.

[14] H. Zang, J. Lou, S. Jiao, H. Li, Y. Du, J. Wang, *J. Mol. Liq.* **2021**, *330*, 115667.

[15] K. Wang, Y. Xiao, C. Wu, Y. Feng, Z. Liu, X. Zhu, H. Zang, *Carbohydr. Res.* **2023**, *524*, 108742.

[16] K. W. Omari, L. Dodot, F. M. Kerton, *ChemSusChem* **2012**, *5*, 1767-1772.

[17] H. Zang, Z. Liu, C. Wu, Y. Chang, X. Zhu, X. Zhu, M. Yan, *Sustain. Chem. Pharm.* **2024**, *37*, 101388.

[18] J. Zhao, Z. Guo, C. Marcus Pedersen, L. Jia, S. Jia, X. Hou, Y. Wang, Y. Qiao, *J. Mol. Liq.* **2024**, *413*, 126006.

[19] J. G. Pereira, J. M. J. M. Ravasco, I. Marques, P.-Y. Kao, P.-Y. Li, R. F. A. Gomes, L. Bustillo, T. Rodrigues, C. A. Mateus Afonso, A. Peixoto, Y.-C. Lin, V. D. B. Bonifácio, *Green Chem.* **2025**, *27*, 1740-1746.

[20] K. Yamazaki, N. Hiyoshi, A. Yamaguchi, *ChemistryOpen* **2023**, *12*, e202300148.
